# Congenic expression of poly-GA but not poly-PR in mice triggers selective neuron loss and interferon responses found in *C9orf72* ALS

**DOI:** 10.1007/s00401-020-02176-0

**Published:** 2020-06-19

**Authors:** Katherine D. LaClair, Qihui Zhou, Meike Michaelsen, Benedikt Wefers, Monika S. Brill, Aleksandar Janjic, Birgit Rathkolb, Daniel Farny, Mikolaj Cygan, Martin Hrabe de Angelis, Wolfgang Wurst, Manuela Neumann, Wolfgang Enard, Thomas Misgeld, Thomas Arzberger, Dieter Edbauer

**Affiliations:** 1 German Center for Neurodegenerative Diseases (DZNE), Munich, 81377 Munich, Germany; 2 Munich Cluster for Systems Neurology (SyNergy), 80336 Munich, Germany; 3 Institute of Neuronal Cell Biology, Technische Universität München, 80802 Munich, Germany; 4 Department Biology II, Anthropology and Human Genomics, Ludwig-Maximilians-University Munich, 82152 Martinsried, Germany; 5 German Mouse Clinic, Institute for Experimental Genetics, German Research Center for Environmental Health, Helmholtz Zentrum München, Neuherberg, Germany; 6 Institute of Molecular Animal Breeding and Biotechnology, Gene Center, Ludwig-Maximilians-University Munich, 81377 Munich, Germany; 7 German Center for Diabetes Research (DZD), Ingolstädter Landstr. 1, 85764 Neuherberg, Germany; 8 Chair of Experimental Genetics, School of Life Science Weihenstephan, Technische Universität München, Alte Akademie 8, 85354 Freising, Germany; 9 German Center for Neurodegenerative Diseases (DZNE), Tübingen, Germany; 10 Department of Neuropathology, University of Tübingen, Tübingen, Germany; 11 Center for Neuropathology and Prion Research, Ludwig-Maximilians-University Munich, 81377 Munich, Germany; 12 Department of Psychiatry and Psychotherapy, University Hospital, Ludwig-Maximilians-University Munich, 80336 Munich, Germany; 13 Graduate School of Systemic Neurosciences (GSN), Ludwig-Maximilians-University Munich, 81377 Munich, Germany

**Keywords:** *C9orf72*, ALS, FTD, Neurodegeneration, Mouse model, Microglia, Interferon

## Abstract

Expansion of a (G_4_C_2_)_*n*_ repeat in *C9orf72* causes amyotrophic lateral sclerosis (ALS) and frontotemporal dementia (FTD), but the link of the five repeat-encoded dipeptide repeat (DPR) proteins to neuroinflammation, TDP-43 pathology, and neurodegeneration is unclear. Poly-PR is most toxic in vitro, but poly-GA is far more abundant in patients. To directly compare these in vivo, we created congenic poly-GA and poly-PR mice. 40% of poly-PR mice were affected with ataxia and seizures, requiring euthanasia by 6 weeks of age. The remaining poly-PR mice were asymptomatic at 14 months of age, likely due to an 80% reduction of the transgene mRNA in this subgroup. In contrast, all poly-GA mice showed selective neuron loss, inflammation, as well as muscle denervation and wasting requiring euthanasia before 7 weeks of age. In-depth analysis of peripheral organs and blood samples suggests that peripheral organ failure does not drive these phenotypes. Although transgene mRNA levels were similar between poly-GA and affected poly-PR mice, poly-GA aggregated far more abundantly than poly-PR in the CNS and was also found in skeletal muscle. In addition, TDP-43 and other disease-linked RNA-binding proteins co-aggregated in rare nuclear inclusions in the hippocampus and frontal cortex only in poly-GA mice. Transcriptome analysis revealed activation of an interferon-responsive pro-inflammatory microglial signature in end-stage poly-GA but not poly-PR mice. This signature was also found in all ALS patients and enriched in *C9orf72* cases. In summary, our rigorous comparison of poly-GA and poly-PR toxicity in vivo indicates that poly-GA, but not poly-PR at the same mRNA expression level, promotes interferon responses in *C9orf72* disease and contributes to TDP-43 abnormalities and neuron loss selectively in disease-relevant regions.

**Electronic supplementary material:**

The online version of this article (10.1007/s00401-020-02176-0) contains supplementary material, which is available to authorized users.

## Introduction

Amyotrophic lateral sclerosis (ALS) and frontotemporal dementia (FTD) are fatal neurodegenerative diseases with overlapping clinical symptoms, neuropathological findings and genetic causes. Predominantly cytoplasmic aggregates of the nuclear RNA-binding protein TDP-43 are a key feature that correlates with regional neurodegeneration and neuroinflammation [37, 47]. About 10% of all ALS/FTD patients carry a pathogenic (G_4_C_2_)_*n*_ repeat expansion in the *C9orf72* gene. The sense and antisense repeat transcripts are translated in all reading frames into five aggregating dipeptide repeat proteins (DPRs: poly-GA, -GP, -GR, -PA and -PR) in an unconventional process termed repeat-associated non-ATG (RAN) translation [1, 45, 46, 70]. In patients, the DPR proteins co-aggregate in neuronal cytoplasmic inclusions and occasionally in neuronal nuclear inclusions [58]. DPR inclusions are also found in skeletal muscle of 40% of patients [9]. Poly-GA, translated from the sense strand, forms the most abundant DPR inclusions in *C9orf72* carriers, found in all neocortical regions, hippocampus and cerebellum, and to a lesser degree in subcortical regions, brainstem and the spinal cord [37, 38, 58]. In contrast, poly-PR is translated from the antisense strand and inclusions are about 100-fold less abundant than for poly-GA in patients [38], but it is clearly the most toxic in cellular models [31, 62, 63]. Furthermore, in cellular models, poly-GA expression has been most directly linked to TDP-43 aggregation [28, 29, 48]. In addition, sense and antisense repeat RNA accumulate in ubiquitous nuclear foci that may interact with a plethora of RNA-binding proteins with high affinity for (G_4_C_2_)_*n*_ RNA, e.g., hnRNP H [8], which may affect splicing and gene expression. Finally, the mutant *C9orf72* allele is poorly expressed, resulting in widespread reduction of C9orf72 protein expression by 20–40% [16, 55]. However, even complete *C9orf72* knockout does not cause ALS/FTD like phenotypes in mice [23], arguing against loss-of-function as a major contributor to disease. Importantly, none of the *C9orf72*-specific phenomena clearly correlate with TDP-43 pathology or neuron loss in patients, arguing for complex synergistic effects and/or selective vulnerability [14].

Among BAC transgenic *C9orf72* models that produce both repeat RNA and DPRs, some have shown subtle behavioral phenotypes and neuron loss, but the relative contributions of RNA and DPRs cannot be determined [23, 49, 53]. Only one *C9orf72* BAC line shows an aggressive ALS-like phenotype, but only in a subset of female mice [34]. Models that express translation-incompetent repeat RNA or individual DPRs have begun to unravel their individual contributions in *C9orf72* disease [6, 7, 20, 23, 34, 43, 49, 53, 57, 66–68]. High-level viral expression of (G_4_C_2_)_n_ RNA, poly-GA, poly-GR, or poly-PR cause widespread DPR pathology and variable neurodegeneration by 3–6 months [6, 66–68]. For transgenic lines, homozygous poly-PR-expressing mice so far showed the most severe toxicity due to Purkinje cell loss leading to cerebellar ataxia, but not clearly ALS-like phenotypes [7, 20, 57]. Together, this body of work suggests that poly-GA, poly-GR, and poly-PR can all be toxic to the CNS in vivo if the expression levels are high enough. However, the expression strategy and repeat lengths are different for each model, precluding rigorous comparison of the relative contributions of specific DPRs to disease phenotypes and pathomechanisms.

Thus, we generated and characterized novel conditional mouse lines for high-level genomic expression of individual DPR proteins utilizing alternate codon sequences to avoid effects of (G_4_C_2_)_*n*_ RNA toxicity and avoid any potential effects of differential RAN translation efficiency. We focused on the most abundant and the most toxic DPRs, poly-GA and poly-PR, respectively. We show that poly-PR expression caused seizures and ataxia in the absence of detectable neuronal loss in 40% of mice. In contrast, all poly-GA mice developed denervation of neuromuscular junctions and muscle wasting with eventual weight loss requiring euthanasia by 6–7 weeks. We find selective vulnerability of the hippocampus and spinal motoneurons towards poly-GA. Finally, we identify a *C9orf72-*enriched inflammatory signature shared between poly-GA mice and human *C9orf72* ALS patients. The rapid progression of our new poly-GA mouse line and the strong inflammatory component that mimics the ALS spinal cord will allow rapid preclinical studies of poly-GA- and interferon-directed therapeutics.

## Materials and methods

### Generation and breeding of mice

We generated plasmids for conditional expression of DPRs by intersting GFP-GA_175_ and GFP-PR_175_ genes (encoded using non-repeating alternate codons) downstream of a floxed stop cassette (encoding a puromycin resistance gene followed by SV40 poly-adenylation signal) in the pEX CAG stop-bpA vector and electroporated them together with a PhiC31-Integrase expression plasmid into murine RMCE (recombinase-mediated cassette exchange) embryonic stem cells at the Rosa26 safe harbor [22]. The Rosa26 gene itself was knocked out by the neomycin-bpA gene trap during this process. Targeted integration was screened by neomycin selection and Southern blot. Clones with Cre-inducible DPR expression were injected into blastocytes. We identified mouse lines GAstop and PRstop with germ-line transmission and speed backcrossed them to C57BL/6N background until > 98% purity was confirmed using SNP genotyping (Charles River). Crossing GAstop and PRstop mice with Nestin-Cre [61] and subsequent excision of the stop cassette resulted in DPR expression throughout CNS neurons (ROSA26 GFP-GA_175_^+/−^; Nestin-Cre and ROSA26 GFP-PR_175_^+/−^; Nestin-Cre). Relevant endpoint criteria for euthanasia according to national regulations were weight loss before 10 weeks of age or stage II seizures consisting of sustained cramping causing the mouse to fall on its side or back. Mice were killed by brief CO_2_ exposure, followed by decapitation. Tissue was either stored at − 80 °C for biochemistry analysis or formalin fixed for 24 h for immunohistochemistry analysis. For spinal cord tissue, decalcification using 5% formic acid was performed for 48 h.

Animal handling and animal experiments in this study were performed in accordance with the German animal welfare law and approved by the Government of Upper Bavaria, Germany. Mice were housed in standard cages in a pathogen-free facility in a 12-h light/dark cycle with ad libitum access to food and water.

### Rotarod

14-Month-old animals were acclimated to the experimenter for 1 week before training by placing mice on the back of the hand and allowing them to explore for 1 min. Training was performed on Monday, Wednesday and Friday, and animals were acclimated to the test room for 15 min before beginning. The apparatus was cleaned with 70% EtOH and allowed to dry before each run. Mice were placed on the rotarod (RotaRod, TSE Systems) moving at 4 rpm for three 1-min runs per day, each followed by a 10-min break. If mice fell off the rod, they waited at the bottom of the apparatus for the full minute. The following week, mice were tested for their latency to fall off the rotarod as it increased speed up to 60 rpm over 1 min. Each mouse again performed three testing trials each followed by 10-min breaks.

### Immunohistochemistry and immunofluorescence

Immunohistochemistry and immunofluorescence was performed on 5-µm paraffin-embedded tissue sections as described previously [37] using the following antibodies: Alyref (ab6141, Abcam 1:50), ChAT (AB144P, Millipore 1:200), GFP (632381, Takara 1:250), Fus (A300-292A, Bethyl Labs 1:100), Hnrnpa1 (sc-32301, Santa Cruz 1:50), Hnrnph (A300-511A, Bethyl Labs 1:500), Iba1 (091-19741, Wako, 1:500 and ab5076, Abcam, 1:500), Map2 (M1406, Sigma 1:250), NeuN (ab177487, Abcam, 1:1000), nucleolin (ab50279, Abcam 1:30), p62 (PM045, MBL 1:1000), poly-GA (1A12, mouse, purified and biotinylated, 1:500) [37], poly-PR (32B3, mouse purified and biotinylated, Helmholtz Zentrum 1:1000) [58], Psmc4 (A303-850A, Bethyl Labs 1:250), Alexa Fluor 594 anti-Sfpq (A301-321A, Bethyl Labs 1:100), TDP-43N-terminal (10782-2-AP, Proteintech 1:500), TDP-43 p409/410 (1D3, gift from Manuela Neumann 1:500), TDP-43 p409/410 (CAC-TIP-PTD-P02, Cosmo 1:500), TDP-43 p409/410 (CAC-TIP-PTD-M01, Cosmo 1:1000), TDP43 p409/410 (3655, gift from Leonard Petrucelli 1:500), TDP-43 pS403/404 (CAC-TIP-PTD-P05, Cosmo 1:500), TDP-43 pT153/Y155 (gift from Yuna Ayala 1:300), TDP-43 C-terminal (12892-1-AP, Proteintech 1:100), TDP-43 non-phospho 404–413 (TAR5 6D6, gift from Manuela Neumann 1:5), TDP-43 non-phospho 404–413 (TAR5 2H4, gift from Manuela Neumann 1:5), TDP-43 N-terminal (10782-2-AP, Proteintech 1:500), TDP-43 p409/410 (1D3, gift from Manuela Neumann 1:500), TDP-43 p409/410 (CAC-TIP-PTD-P02, Cosmo 1:500), TDP-43 p409/410 (CAC-TIP-PTD-M01, Cosmo 1:1000), TDP43 p409/410 (3655, gift from Leonard Petrucelli 1:500), TDP-43 pS403/404 (CAC-TIP-PTD-P05, Cosmo 1:500), TDP-43 pT153/Y155 (gift from Yuna Ayala 1:300), TDP-43 C-terminal (12892-1-AP, Proteintech 1:100), TDP-43 non-phospho 404–413 (TAR5 6D6, gift from Manuela Neumann 1:5), TDP-43 non-phospho 404–413 (TAR5 2H4, gift from Manuela Neumann 1:5), Tubulin β 3 (801208, BioLegend 1:200). Nucleolin immunofluorescence required 1–5 min treatment with 50 µg/mL protease K prior to citrate antigen retrieval. Alexa Fluor 555 Mouse anti-β-Tubulin III (BD Pharmingen 560,339 1:200) and Bungarotoxin Alexa Fluor 647 (Thermo Fisher B35450 1:500) were used to stain motor axons and acetyl-choline receptors at the NMJ, respectively.

### Immunofluorescence and histological quantifications

Automated quantification of immunofluorescence was performed using CellProfiler (v. 3.0.0) software. For poly-PR quantification, images from sagittal sections approximately 100 µm from the midline were taken covering the entire cortex of each animal. DAPI-stained nuclei and poly-PR aggregates were identified and counted, and the % of neurons with poly-PR inclusions was reported for each animal. For neuron quantification in brain and spinal cord, 10 × scanning images were taken of the entire region on each slide (cortex, hippocampus, or spinal cord). NeuN-positive cells were identified using CellProfiler and divided by the area of the tissue section measured. Because the loss of NeuN can occur in intact neuronal nuclei [65], we also verified that there were no large nuclei with neuronal morphology in the CA fields of the hippocampus stained with DAPI but not NeuN. We used NeuN staining only to assist with semi-automated detection of neurons vs glia in our immunofluorescence staining. Choline acetyltransferase (ChAT)-positive cells with motor neuron morphology in the anterior horn were quantified manually on sections at 1-mm intervals spanning the whole spinal cord. For quantification of the denervated neuromuscular junctions (NMJs), confocal image stacks were recorded using an LSM810 confocal laser scanning system (Carl Zeiss) with 25 × water immersion objective. Innervated or denervated single NMJs in the triangularis sterni and quadriceps muscles were classified by an investigator blinded to genotype.

### Western blotting and ELISA

Tissues were collected on ice and lysed in 10 volumes of RIPA buffer with protease inhibitor and phosphatase inhibitor supplements. Tissue was centrifuged at 1000×*g* to remove cell debris but preserve DPR aggregates in the supernatant fraction. Protein concentration was determined by BCA assay. For western blot, 20 µg of protein was loaded per lane on 10% SDS-acrylamide gels and separated by gel electrophoresis in Tris–glycine–SDS buffer, followed by transfer to PVDF membranes. Membranes were blocked with 5% iBlock (Thermo Fisher, T2015) in TBS–Triton x-100, or for detection of phosphorylated proteins in 5% bovine serum albumin (Thermo Fisher). We performed western blotting using the following antibodies: GFP (632381, Takara 1:1000), Stat1 (#9172, Cell Signaling 1:1000), Tubb3 (TUJ1, Biolegend 1:1000), Dlg4 (PSD-95) (K28/74, Neuromab 1:1000), Syp (Sy38, Millipore 1:1000), Arc (sc-17839, Santa Cruz 1:200), pStat1 Tyr701 (#7649, Cell Signaling 1:500), Stat2 (D9J7L, Cell Signaling 1:1k), pStat2 Y690 (ab53132, Abcam 1:500), Stat3 (#79D7, Cell Signaling 1:1000), p-Stat3 Tyr705 (#9145, Cell Signaling 1:1000), calnexin (SPA-860F, Enzo 1:7000). After washing in TBS-Tx, membranes were probed with anti-mouse or anti-rabbit HRP conjugated antibodies at 1:5000 in 5% iBlock. HRP-bound proteins were detected with ECL Western blotting substrate (Pierce, 32106). Cytokine ELISAs were performed according to the manufacturer’s instructions using Legend Max mouse TNFα and IFNγ ELISA kits (Biolegend) and read on a QuickPlex SQ 120 (Mesoscale).

### Hematological analysis

Blood samples were collected by final blood withdrawal under isoflurane anesthesia by puncturing the retrobulbar vein plexus with a 1-mm-diameter glass capillary (Hirschmann). Blood was collected in EDTA-coated sample tubes (Kabe Labortechnik) and an amount of 50 µl determined by an end-to-end capillary (Kabe Labortechnik) was diluted 1:5 with Cell Pack buffer (Sysmex) and stored at room temperature until analysis. To evaluate peripheral blood cell counts including automated differential white blood counts samples were mixed thoroughly by inverting the tube 3–4 times and analyzed using a Sysmex XT 2000iV hematology analyzer in the “capillary mode” with the predefined mouse settings within 6 h after sample collection and dilution.

### RNA isolation and library preparation

Tissues were homogenized in Trizol using homogenizer (Precellys). Total RNA was isolated using Direct-zol-96 RNA isolation kit (ZymoResearch) following the manufacturer’s instructions. RNA quantity and quality were controlled on Agilent 2100 BioAnalyzer using Agilent RNA 6000 Nano Kit.

Reverse transcription was performed at 42 °C for 90 min with a reaction consisting of 25 units Maxima H- enzyme (Thermo Fisher), 2 × Maxima H-Buffer (Thermo Fisher), 2 mM each dNTPs (Thermo Fisher), 4 µM template-switching oligo-(IDT), 4 µM barcoded oligo-dT primers (IDT), and 10 ng of RNA, in a final reaction volume of 10 µL. Following RT, the cDNA was pooled and cleaned using SPRI beads, and then eluted with 17 µL of DNase/RNase-Free Distilled Water (ThermoFisher). Residual primers were digested with Exonuclease I (Thermo Fisher) at 37 °C for 20 min, with a subsequent heat inactivation step at 80 °C for 10 min. Pre-amplification was then performed by adding 25 µL of KAPA HiFi HotStart ReadyMix (2x, KAPABIOSYSTEMS) and 0.33 µM SINGV6 primer (IDT) for a final reaction volume of 50 µL. The PCR was cycled as follows: 98 °C for 3 min for initial denaturation followed by 11 cycles of 98 °C for 15 s, 65 °C for 30 s, 68 °C for 4 min. Final elongation was performed at 72 °C for 10 min.

Following pre-amplification, the sample was cleaned using SPRI beads (1:0.8) and then eluted with 10 µL of DNase/RNase-free distilled water (ThermoFisher). The cDNA was quantified using the Quant-iT PicoGreen dsDNA Assay Kit (Thermo Fisher) and size distributions were checked on high-sensitivity DNA chips (Agilent Bioanalyzer). After passing the quantity and quality controls, 0.8 ng of pre-amplified cDNA was used to construct Nextera XT libraries (Illumina). The library PCR used custom P5 primer (P5NEXTPT5, IDT) to enrich for the 3′ ends. The library was then size selected using a 2% Agarose E-gel (Life Technologies) from 300–800 bp.

### qPCR

qPCR was performed as described previously [69]. qRT-PCR was performed using CFX384 Real‐Time System (Bio‐Rad) with Taqman technology. For GFP expression in the brain, primers and probes were designed (IDT) for the GFP region. Primer 1: GCACAAGCTGGAGTACAACTA, Primer 2: TGTTGTGGCGGATCTTGAA, Probe/56FAM/AGCAGAAGA/ZEN/ACGGCATCAAGGTGA/3IABkFQ/. Signals were normalized to GAPDH levels according to ΔΔCT method and levels are shown relative to control. For GFP expression in the muscle, Taqman probes (ThermoFisher) were used against GFP (Mr04097229_mr) and Atp5a1 (Mm00431960_m1) with Sybr fluor. GFP signals were normalized to Atp5a according to the ΔΔCT method and levels are shown relative to control.

### Sequencing and primary data processing

Final libraries were paired-end sequenced on a high output flow cell of a HiSeq 1500 (Illumina), with the following sequencing setup: 16 bases for the cellular barcode and UMI, eight bases for the i7 barcode, and 50 bases for the cDNA read. Libraries were sequenced to a depth of ~ 10 million reads per sample. Raw fastq files were checked using FastQC (v 0.10.1) and then processed using zUMIs (v 0.0.6) with default settings and a provided barcode list. The data were mapped to the mouse genome (mm10) and gene annotations were obtained from Ensembl (GRCh38.89).

### Data analysis

The count tables (from tissue mean counts 2.4 M, range 0.225–28 M; from isolated microglia average counts 250 K, range 153–678 K; samples with less than 100 K counts were excluded) were analyzed using the DESeq2 pipeline in R comparing transgenic mice and littermate controls for each region and age separately [36]. The genotype of all mice was confirmed (and corrected) using the GFP-(GA)_175_ and GFP-(PR)_175_ transcript. The number of samples per group was 3 weeks (7 control vs 3 GA-Nes), end-stage (5 control vs 5 GA-Nes), isolated microglia at end-stage (3 control vs 3 GA-Nes), affected (5 control vs 5 PR-Nes), and asymptomatic (3 control vs 5 PR-Nes). All significant hits (*p* < 0.05 adjusted for multiple testing for each pairwise comparison) are listed in Table S2. All publicly available RNAseq data from TargetALS data were accessed in February 2020. We included all samples from patients with ALS and controls without CNS co-morbidity and with *C9orf72*-genotype information available. Reads were aligned to GRCh38 using STAR aligner b2.4.2a [12] by the New York Genome Center and further processed by us using SummarizeOverlaps in R. Count tables were analyzed using DESeq2 [36] in a linear model accounting for gender as a confounder in the cerebellum (14 controls, 108 ALS, and 30 *C9orf72* ALS) and frontal cortex (16 controls, 90 ALS, and 26 *C9orf72* ALS), and differential gene expression was analyzed between the relevant groups. A model accounting for gender and anatomical sub-regions was used for spinal cord (cervical: 13 controls, 86 ALS, 25 *C9orf72* ALS; thoracic: eight controls, 44 ALS, 7 *C9orf72*; lumbar: 11 controls, 77 ALS, 19 *C9orf72* ALS) and motor cortex (lateral: 13 controls, 77 ALS, 21 *C9orf72* ALS; medial: 15 control, 78 ALS and 18 *C9orf72* ALS). The overall LFC for the groups was used for the correlation analysis.

Gene ontology analysis (biological process) was done using the clusterProfiler package in R. Only groups with more than five significant genes were analyzed. Figures show a manual selection of the most significant and least overlapping categories to best represent the key pathways. The full list of significant GO terms is shown in Tables S3 and S5. Concordantly regulated genes were defined by comparing averaged significant up-/down-regulation from all analyzed regions in GA-Nes or PR-Nes mice and ALS patients.

Kmeans clustering was done using default parameters with pheatmap (1.0.12) in R. Pearson’s correlation of gene expression was done using pairwise-complete observations with identical Entrez gene names. We used the log2-fold expression changes of significant genes from the AD and SOD1 mouse models (padj < 0.05) [26], and the p25 mouse model in reference [40] (cZ > 3.0902). For the microglia clusters, we used all genes listed in the supplemental data of reference [19]. The actual number of genes used is indicated in the figure legends.

### Statistics

Statistical analyses other than for RNAseq data (described separately above) were performed using GraphPad Prism 7.02. We used two-tailed *t* tests to compare two groups, and ANOVAs with Greenhouse–Geisser correction to compare more than two groups. Data are reported in the figure legends with (mean ± standard deviation) and effect size (*η*^2^, the between-group sum-of-squares divided by the total sum-of-squares). Confidence intervals are reported instead of effect size for non-parametric tests. Data distributions were checked for normality by the Shapiro–Wilk test and homogeneity of variances was checked by the *F* test or the Brown–Forsythe test. When these were violated, non-parametric tests were used (Mann–Whitney). Individual points for immunoblots represent the average of at least two independent replicates per biological sample. Power analyses were performed with GPower 3.1.

## Results

### Poly-GA expression causes progressive weakness, while poly-PR triggers ataxia and seizures with high expression and no symptoms with low expression

To assess only the contribution of the DPR proteins in the absence of G_4_C_2_ RNA toxicity, we used non-repeating alternate codons to individually express poly-GA and poly-PR in the absence of (G_4_C_2_)_*n*_ repeats as in previous models [41, 57]. We generated conditional single transgenic DPR expressing mice by targeted insertion of a GFP-GA_175_ or GFP-PR_175_ expression construct driven by the strong CAG promoter with a LoxP-flanked stop cassette at the Rosa26 safe harbor locus (Fig. S1a). To allow CNS-wide neuronal expression we crossed these mice with the well-characterized Nestin-Cre driver line (Fig. S1a, b) [61] creating double transgenic mice (GA-Nes and PR-Nes). GA-Nes and PR-Nes mice were born at Mendelian ratios (23.5% *χ*^2^ = 2.327, *df* = 3, *p* = *0.5075n* ≥ 44/group and 23.5% *χ*^2^ = 6.647, *df* = 3, *p* = *0.084n* ≥ 24/group, respectively).

**Fig. 1:**
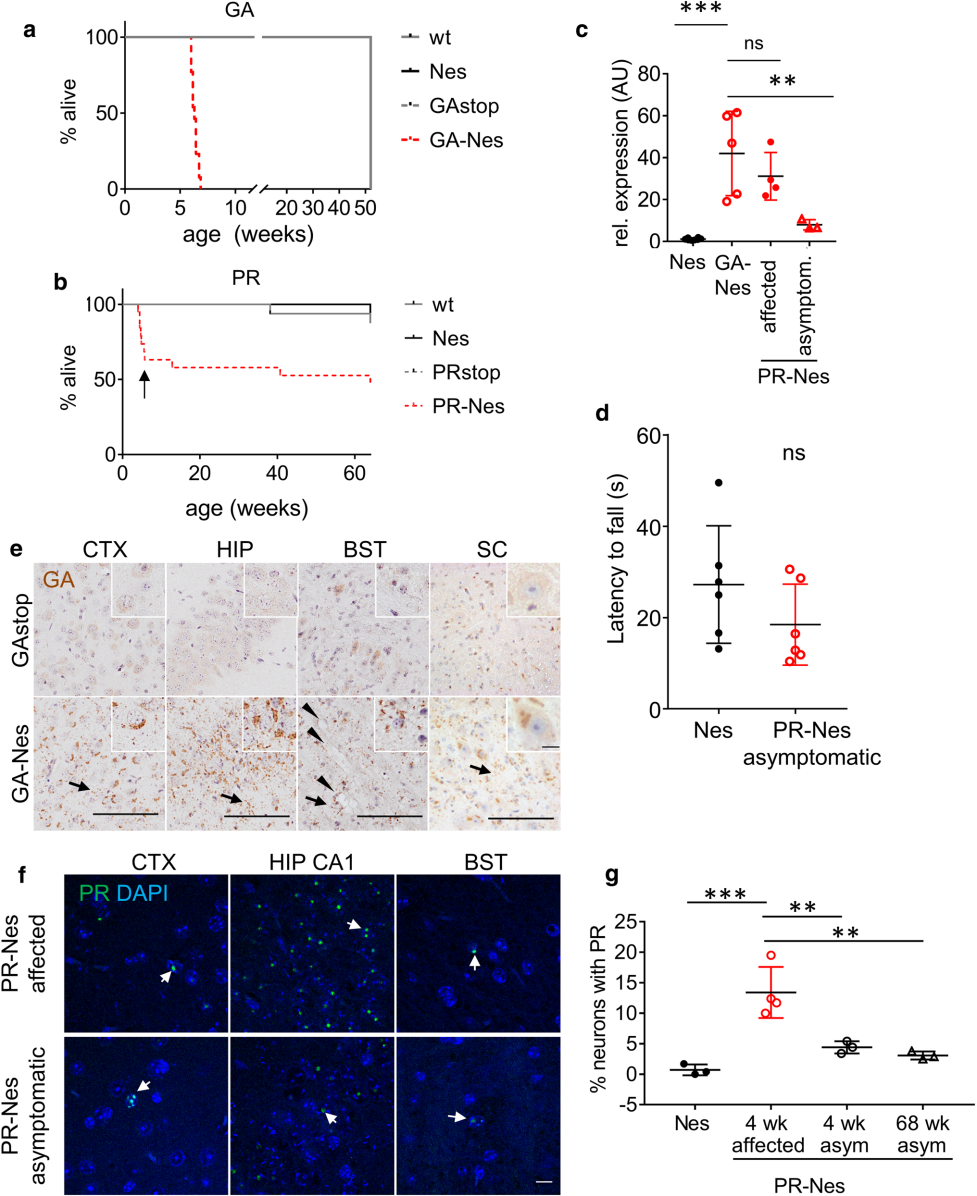
All GA-Nes mice and a subset of PR-Nes mice reach the humane endpoint rapidly, while remaining PR-Nes mice downregulate transgene expression and live normal life spans without motor deficits. **a** All GA-Nes mice reach the humane endpoint by 7 weeks of age due to weakness, while all animals of control genotypes remain healthy until the end of the study at 52.1 weeks. Mantel–Cox *χ*^2^ = 44.35, *p* < 0.001. Median endpoint wt = 52.1 (*n* = 9), Nes = 52.1 (*n* = 5), GAstop = 52.1 (*n* = 8), GA-Nes = 6.3 (*n* = 13). **b** 40% of PR-Nes mice reach the humane endpoint by 5 weeks of age due to seizure and ataxia and make up the affected group (arrow). The other mice remain asymptomatic. Mantel–Cox *χ*^2^ = 11.32, *p* = 0.01. Median endpoint wt = 69.9 (*n* = 16), Nes = 70 (*n* = 16), PRstop = 70.1 (*n* = 11), PR-Nes = 69.3 (*n* = 19). Study duration: 68 weeks. **c** Expression of poly-GA and poly-PR mRNA is comparable between GA-Nes and affected PR-Nes mice, but not asymptomatic PR-Nes (AU = arbitrary units).[ANOVA F(3,16) = 16.2 *p* < 0.001 *η*^2^ = 0.753, Dunn’s post hoc Nes (1.1 ± 0.51) vs GA-Nes (42 ± 20.1) ***p < 0.001, GA-Nes (42 ± 20.1) vs affected PR-Nes (31.1 ± 11.3) ns *p* = 0.365, GA-Nes(42 ± 20.1) vs asymptomatic PR-Nes (8.0 ± 2.4) **p = 0.002]. Nes *n* = 8, GA-Nes *n* = 5, affected PR-Nes *n* = 4, asymptomatic PR-Nes *n* = 3. **d** Rotarod assay shows no significant difference in latency to fall (s = seconds) at 14 months of age [*t*(6) = 1374, Nes (27.2 ± 12.9) vs asymptomatic PR-Nes (18.43 ± 8.9) *p* = 0.199 *η*^2^ = 0.159] *n* = 6 per group. Power to detect *η*^2^ > 0.5 = 52%. **e** Representative images of GA-GFP immunohistochemistry staining (brown) at 3 weeks in cortex (CTX), hippocampus CA2 (HIP), brainstem (BST) and spinal cord (SC), including motor neurons (inset shown). Poly-GA aggregates are also frequent in the neuropil (arrows). Counterstain hematoxylin. Extracellular vacuoles are visible near axon tracts in the brainstem (arrowheads). Scale bar = 100 µm, insets = 10 µm. **f** Representative images of PR-GFP immunofluorescence shows predominantly low-abundance nuclear aggregates (arrows) in the neocortex (CTX) and brainstem (BST), but higher abundance in the CA1 region of the hippocampus (HIP CA1), especially in affected mice, scale bar = 10 µm. For visibility in printed versions, the intensity of the green channel was doubled in all images. Closer views of PR aggregates are shown in Fig. 2i,j. **g** Automated quantification of the % of neurons with poly-PR inclusions in the neocortex of affected and asymptomatic (asym) PR-Nes and control (Nes) mice [ANOVA F(3,9) = 17.7, *p* < 0.001 *η*^2^ = 0.855; Holm–Sidak post hoc Nes(0.7 ± 0.889) vs 4 weeks affected (13.4 ± 4.2) ***p = 0.001, 4 weeks affected (13.4 ± 4.2) vs 4 weeks asym (4.4 ± 1.0) **p = 0.005, 4 weeks affected (13.4 ± 4.2) vs 68 weeks asym (3.1 ± 0.7) **p = 0.002]. 4 weeks Nes *n* = 3, 4 wk affected *n* = 4, 4 weeks asym *n* = 3, 68 weeks asym *n* = 3

We observed progressive motor deficits and early lethality in all GA-Nes mice and a subset of PR-Nes mice. All GA-Nes mice showed abnormal gait and impaired motor function from 5 weeks (S. video 1), and by 6–7 weeks mice had severe muscle weakness but not paralysis (S. video 2). GA-Nes mice were smaller than control littermates beginning around 4 weeks of age, and later even began to lose weight, at which time we were required to euthanize them in accordance with national animal welfare laws. All GA-Nes mice reached this end-stage point before 7 weeks, resulting in a sharp drop in the Kaplan–Meier curve (Fig. 1a), and providing food on the floor of the cage did not improve survival. In contrast, about 40% of PR-Nes mice also failed to thrive and around 4–5 weeks of age developed seizures that were criteria for euthanasia (Fig. 1b). These mice also showed poor coordination reminiscent of ataxia and fell frequently to the side without apparent muscle weakness (Supp. video 3). The remaining PR-Nes mice that did not meet endpoint criteria early on did not develop seizures later in life, gained weight normally and had normal motor function until the end of the study at 68 weeks (Supp. video 4). The rotarod assay revealed no significant motor deficits in these mice even at 14 months (Fig. 1d). Due to these radically different phenotypes, also seen to a lesser extent upon viral poly-PR expression [68], we separated PR-Nes mice into affected (4 weeks) and asymptomatic (4 weeks and 68 weeks) groups for the remaining studies. qPCR analysis showed similar transgene expression at the mRNA levels in the cortex of GA-Nes mice and affected PR-Nes mice, but significantly reduced transgene expression in asymptomatic PR-Nes mice (Fig. 1c). Genomic PCR confirmed that asymptomatic PR-Nes still maintained the 175-repeat length suggesting transcriptional repression of the synthetic construct (Fig. S1c).

To elucidate the differential phenotypes in GA-Nes and PR-Nes mice, we first compared DPR expression patterns in both lines using immunostaining. As expected, in the absence of Cre expression (GAstop), no poly-GA expression was detected (Fig. 1e). Consistent with the pan-neuronal expression of the Nestin-Cre driver, poly-GA formed frequent neuronal cytoplasmic inclusions (NCI) and neuritic aggregates in the vast majority of neurons throughout the CNS of GA-Nes mice (Fig. 1e). Combined fluorescent in situ hybridization (FISH) to label the transgene mRNA and immunostaining of Cnp1, GFAP and Iba1 revealed transgene expression in a subset of oligodendrocytes and astrocytes, but not in microglia (Fig. S1d–f). Importantly, despite similar mRNA levels in GA-Nes and affected PR-Nes mice, poly-PR inclusions were infrequent in the cortex and brainstem (Fig. 1f) and not found in the spinal cord. However, in the hippocampus, most neurons had at least one nuclear inclusion in affected PR-Nes mice, while far fewer hippocampal neurons contained inclusions in asymptomatic PR-Nes mice (68 weeks) (Fig. 1f). Quantification of poly-PR in the cortex showed that inclusions in asymptomatic PR-Nes mice were already reduced at 4 weeks, and this persisted in asymptomatics at 68 weeks (Fig. 1g). By immunoblotting with a GFP antibody, poly-PR (aggregated or soluble) was below the detection level even in affected mice, in contrast to abundant aggregated poly-GA visible in the gel pocket (Fig. S1g). Poly-PR aggregates were barely detectable when 25 times more brain extracts than necessary for poly-GA detection were loaded on a filter trap assay (Fig. S1h). Our findings are consistent with the abundant poly-GA and scarce poly-PR inclusion pathology observed in *C9orf72* patients [38, 58], though both are more frequent than observed in patients. Overall, the comparison between these congenic lines shows consistent progressive weakness in poly-GA mice that more closely resembles ALS than the ataxia and seizures found in poly-PR mice, a subset of which escape toxicity completely by reducing transgene expression.

### Selective neuron loss and microglial infiltration in the hippocampus of poly-GA but not poly-PR mice

**Fig. 2:**
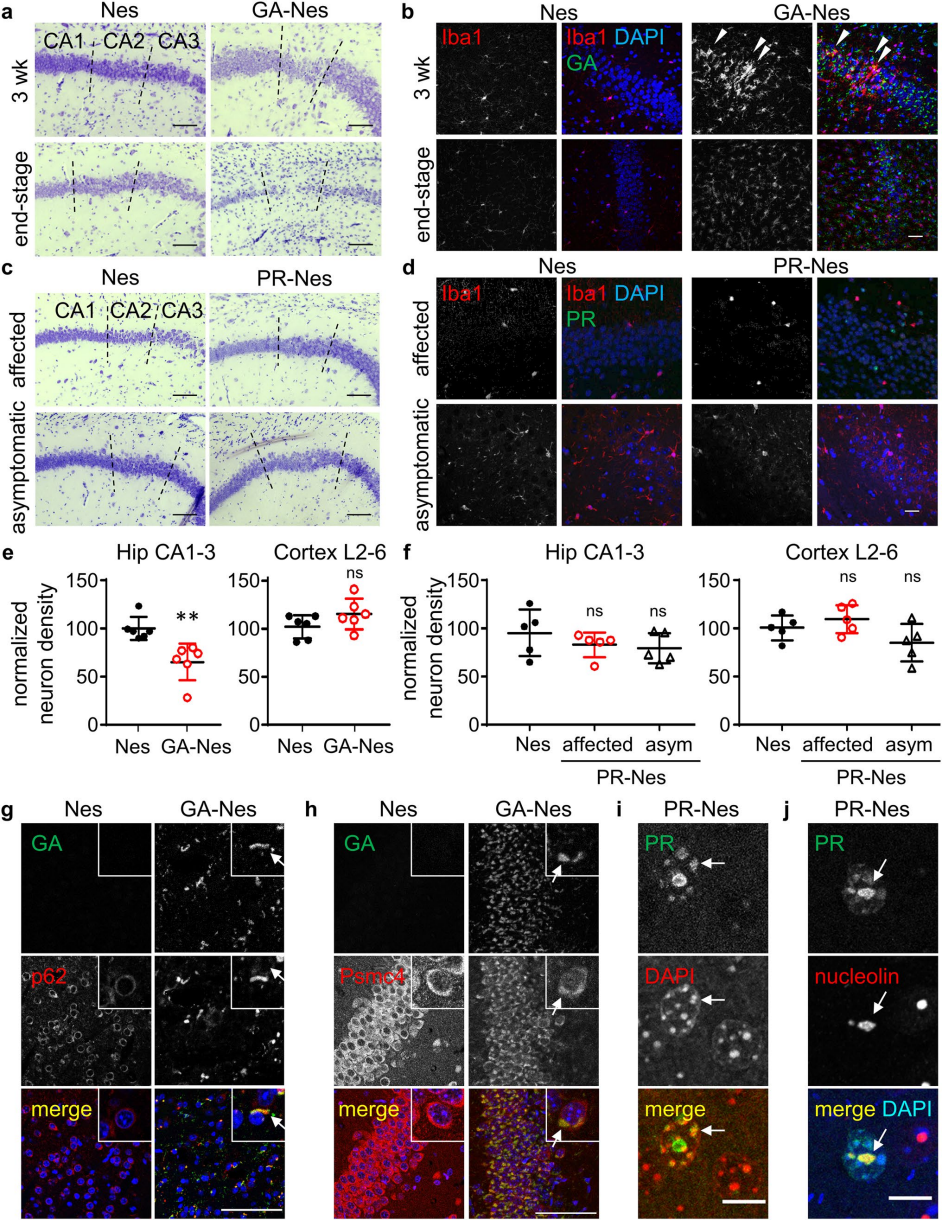
Poly-GA leads to early microglial infiltration in the hippocampus preceding neuron loss, while poly-PR does not. **a** Nissl stains show thinning of the CA neuronal layers in GA-Nes mice beginning at 3 weeks, causing nearly complete ablation of the CA2 by end-stage in contrast to Nes control mice, scale bar = 100 µm. For wider views see Fig. S2a. **b** Clusters of activated amoeboid microglia (Iba1, red) infiltrate the CA2 in GA-Nes mice at 3 weeks of age, despite GA aggregates (GFP, green) in virtually all surrounding neurons. Microglia return to ramified morphology and are homogeneously distributed in the CA2 and the surrounding region by end-stage, scale bar = 25 µm. **c** Nissl stains show no thinning of the CA neuronal layers in affected or asymptomatic PR-Nes mice (right column) compared to Nes controls (left column), scale bar = 100 µm. For wider views see Fig. S2b. **d** Immunofluorescence shows normal ramification and no clustering of microglia (Iba1) in the hippocampus of PR-Nes affected or asymptomatic mice compared to Nes controls despite the presence of PR aggregates (GFP, green), scale bar = 25 µm. **e** GA-Nes mice show significant neuron loss (NeuN positive cells) in the CA1-3 of the hippocampus [Welch’s *t*(8.37) = 3.77, **p = 0.005 *η*^2^ = 0.629, Nes (99.7 ± 11.9) GA-Nes(65 ± 19.1)], but not in the neocortical layers (L2-6) [*t* test *t*(10) = 1.63, ns *p* = 0.135, *η*^2^ = 0.21, Nes (102 ± 12.1) GA-Nes(115 ± 16)]. *n* = 6/group. **f** No neuron loss (NeuN  positive cells) in the hippocampus or neocortex of affected or asymptomatic (asym) PR-Nes mice [hippocampus: Kruskal–Wallis = 1.82, *p* = 0.427, Nes (95.4 ± 24.1, CI 65.5–125) affected PR-Nes (83.2 ± 12.7, CI 67.5–98.9) asymptomatic PR-Nes (79.8 ± 15.7, CI 60.3–99.3); neocortex: ANOVA *F*(2,12) = 0.307, *p* = 0.084 *η*^2^ = 0.338, Nes (101 ± 12.8) affected PR-Nes (110 ± 14.7) asymptomatic PR-Nes (85 ± 19.4)]. *n* = 5/group. **g** Double immunofluorescence shows colocalization (arrows) of poly-GA aggregates (GFP) with p62 (Sqstm1) in the neocortex (scale bar = 100 µm). Insets (30 × 30 µm) provide a close view of single neurons. DAPI (blue). **h** Double immunofluorescence shows colocalization (arrows) of poly-GA aggregates (GFP) and Psmc4 in the CA1 of the hippocampus at 3 weeks (scale bar = 100 µm). Insets (30 × 30 µm) provide a close view of single neurons. DAPI (blue). **i** Double immunofluorescence shows colocalization (arrows) of poly-PR (GFP, green) and heterochromatin (DAPI, red) in the neocortex of an affected PR-Nes mouse. This colocalization was also observed in asymptomatic mice. Scale bar = 10 µm. j) Representative images of the colocalization (arrows) of PR (GFP, green) and nucleolin (red) in the neocortex of an asymptomatic PR-Nes mouse. This colocalization was also observed in affected mice. DAPI (blue) shows the area of the nucleus. Scale bar = 10 µm

To address the consequences of DPR expression and identify correlates of the motor deficits, we analyzed regional neuron loss and early signs of neurodegeneration and microglial activation in our mouse models. Despite widespread aggregation of poly-GA in the vast majority of CNS neurons, robust microglial infiltration accompanied by mild neuron loss first became apparent in the CA2 of the hippocampus after 3 weeks of age, with nearly complete ablation of the CA2 and thinning of the other CA fields by end-stage (Fig. 2a, b; Fig. S2a). The CA2 is delineated by the end of the compactly layered neurons of the CA1 and the beginning of the larger and more diffusely spaced pyramidal neurons of the CA3. In contrast, both affected and asymptomatic PR-Nes mice showed normal neuron density (Fig. 2c, f, Fig. S2b) and no microglia activation in the hippocampus (Fig. 2d), despite high poly-PR aggregation there (compare Fig. 1f). Surprisingly, infiltration of activated microglia with typical amoeboid morphology in the CA2 region of GA-Nes mice was stronger at 3 weeks of age than at end-stage (Fig. 2b). Quantification at end-stage confirmed significant neuron loss in the whole CA field, even when normalizing to the area of the region in the overall smaller brains (Fig. 2e, Fig. S2a). In contrast, neuron loss was not detected in the cortex of GA-Nes (Fig. 2e, Fig. S2a) or affected PR-Nes mice (Fig. 2f, Fig. S2b).

To address known toxicity mechanisms, we analyzed the subcellular distribution of poly-GA and poly-PR in our mice. Poly-GA co-aggregated with p62/Sqstm1 (Fig. 2g) and the proteasome subunit Psmc4 (Fig. 2h), consistent with cryo-electron tomography data in primary neurons [18]. In PR-Nes mice, poly-PR was restricted to the nucleus and colocalized with heterochromatin (Fig. 2i) and the nucleolus (Fig. 2j) in both affected and asymptomatic mice, as reported in other models [21, 68]. However, despite subcellular localization of both DPRs matching that of other mouse models, only poly-GA led to detectable neuron loss in the hippocampus.

### Selective motoneuron loss and muscle denervation in GA-Nes but not PR-Nes mice

**Fig. 3:**
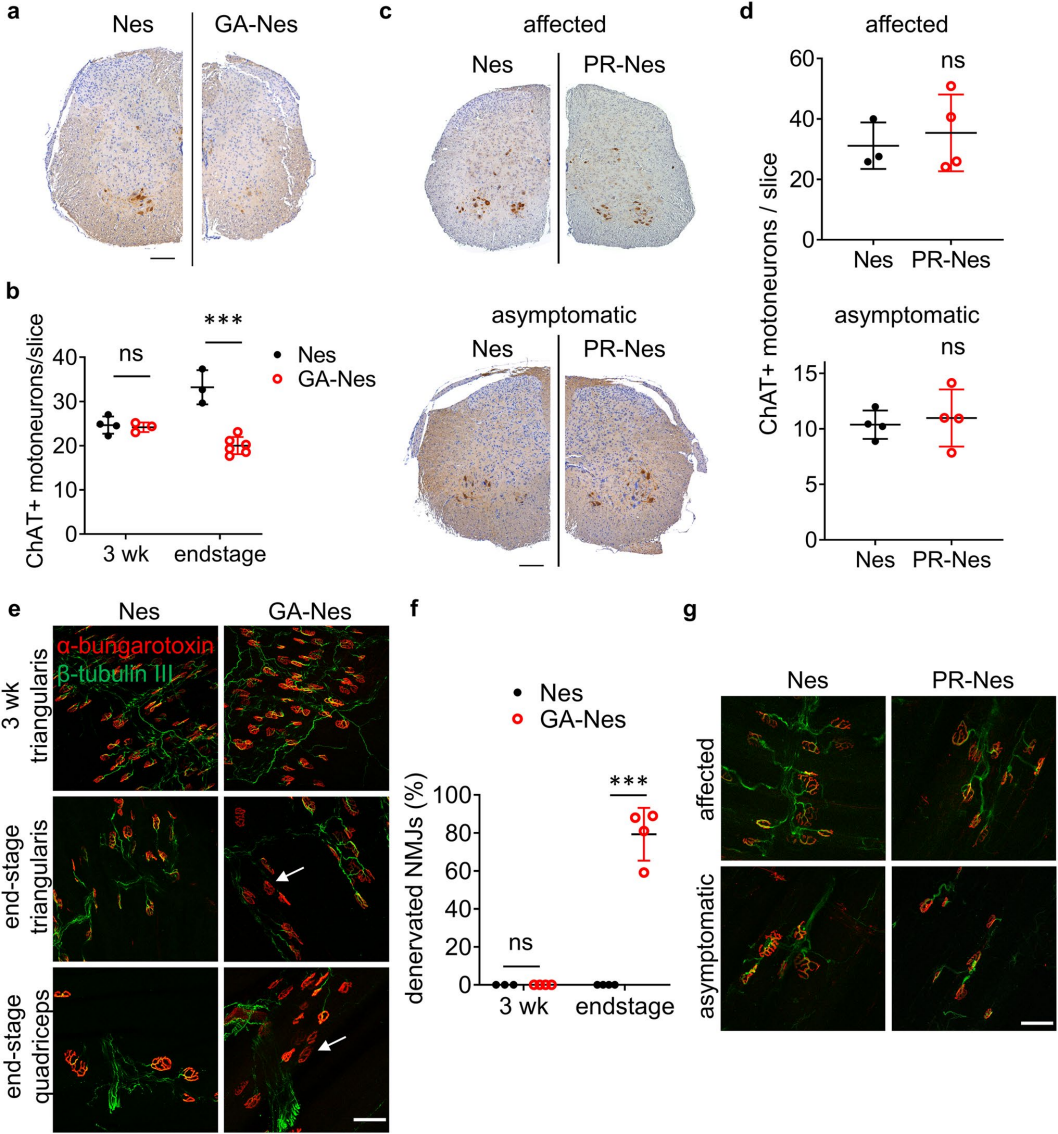
End-stage GA-Nes mice develop selective neurodegeneration in hippocampus and spinal cord and muscle denervation, while PR-Nes mice do not. **a** Immunohistochemical visualization of motoneurons by antibodies against ChAT (brown) in the lumbar spinal cord shows a clear reduction of ChAT expressing cells in the end-stage GA-Nes mouse compared to Nes control. Scale bar = 200 µm. **b** Quantification of average ChAT + motoneurons/slice sampled every 1 mm through the whole spinal cord. GA-Nes mice show significant motor neuron loss by end-stage compared to Nes controls. [Two-way ANOVA interaction (age x genotype) F(1,12) = 28.6, *p* < 0.001 η^2^ = 0.705; 3 wk Nes (24.7 ± 2.0, *n* = 4) vs GA-Nes (24.2 ± 1.1, *n* = 3) ns *p* = 0.96; end-stage Nes (33.2 ± 3.9, *n* = 3) vs GA-Nes (20.1 ± 2.0, *n* = 6) *** *p* < 0.001.] **c** ChAT immunohistochemistry (brown) visualizing motoneurons in the lumbar spinal cord of PR-Nes affected and asymptomatic mice and age-matched Nes mice shows no obvious differences in the number of ChAT expressing cells among all mice. Scale bar = 200 µm. **d** Quantification of average ChAT + motoneurons/slice sampled every 1 mm through the whole spinal cord of affected and asymptomatic PR-Nes mice and age-matched Nes controls shows no significant motor neuron loss in either PR-Nes group. Nes (31.1 ± 7.7, *n* = 3) vs affected PR-Nes (35.4 ± 12.7, *n* = 4) [*t*(5) = 0.505, *p* = 0.635 *η*^2^ = 0.0485]. Nes (10.4 ± 1.3, *n* = 4) vs asymptomatic PR-Nes (11 ± 2.6, *n* = 4) [*t*(6) = 0.416, *p* = 0.692 *η*^2^ = 0.0281]. **e** Representative images of neuromuscular junction (NMJ) immunofluorescence in whole mount (unsectioned) triangularis sterni muscles in 3-week and end-stage GA-Nes mice and Nes littermate controls, and microtome sectioned quadriceps muscles in end-stage GA-Nes mouse and Nes littermate control. Examples of denervated NMJs with loss of β-tubulin III (green) immunostaining within α-bungarotoxin (red) labeled endplates are indicated with arrows. Scale bar = 50 µm. **f** Quantification of % denervated neuromuscular junctions (NMJs) in the triangularis sterni shows that NMJs are unchanged at 3 weeks, but significantly denervated by end-stage in GA-Nes vs Nes littermate controls [Two-way ANOVA interaction (age × genotype) *F*(1,11) = 110, *p* < 0.001 *η*^2^ = 0.91; 3 wk Nes (0.02 ± 0.004, *n* = 3) vs GA-Nes (0.02 ± 0.006, *n* = 4) ns *p* > 0.99; end-stage Nes (0 ± 0, *n* = 4) vs GA-Nes (79.3 ± 13.9, *n* = 4) ***p < 0.001]. **g** Representative images of NMJs in the triangularis sterni muscle of single PR-Nes affected and asymptomatic mice, as well as littermate Nes controls. No denervation was observed in these samples

To investigate the muscle weakness in GA-Nes mice, we also examined spinal cord neurons and neuromuscular junctions. While ChAT-positive motor neurons in the anterior horn of the spinal cord were normally abundant in GA-Nes mice at 3 weeks, they were significantly lost by end-stage (Fig. 3a, b). In contrast, the number of calbindin-positive neurons in the dorsal horn was unchanged even at end-stage (Fig. S2c, d), suggesting selective vulnerability of motor neurons in the spinal cord to poly-GA. Consistent with the lack of muscle wasting in PR-Nes mice, no loss of ChAT-positive neurons in the spinal cord was observed (Fig. 3c, d). We further investigated axonal innervation of neuromuscular junctions in GA-Nes mice. While neuromuscular junctions were fully innervated at 3 weeks in GA-Nes mice, by end-stage most α-bungarotoxin-labeled endplates were denervated, as indicated by loss of β-tubulin III immunostaining in both triangularis sterni (whole-mount preparation [4, 27]) and quadriceps muscles (vibratome sections) (Fig. 3e, f). In contrast, no denervation was observed in the triangularis sterni muscle of PR-Nes affected or asymptomatic mice (Fig. 3g).

Despite the clear correlation between motoneuron loss and weakness, we investigated possible peripheral causes of muscle wasting requiring early euthanasia. Hematological parameters, including white blood cell counts, were not significantly different between GA-Nes and controls (Fig. S3a). Systematic macroscopic and microscopic analyses were conducted in the intestines, pancreas, liver, kidney, adrenal gland, spleen, heart, lung, and thymus. Consistent with the known expression profile of Nestin-Cre [11, 13], GFP staining revealed sparse expression of poly-GA in the kidney and pancreas, as well as in neurons of the myenteric plexus (Fig. S4a). However, H + E staining revealed no signs of necrosis or gross pathological abnormality in these or other organs compared to control littermates, other than smaller size consistent with the overall smaller size of GA-Nes mice (Fig. S4b). In addition, the examination of the intact digestive tract at euthanasia showed processed boli present at regular intervals throughout the entire digestive tract in both GA-Nes and controls arguing against impaired peristalsis or intestinal transit (Fig. S4c). Poly-GA aggregates were also found commonly in the quadriceps muscle (Fig. S4d), again consistent with known expression of Nestin-Cre [13] and with the muscle wasting and denervation observed. FISH and qPCR analysis of the quadriceps confirmed that poly-GA was expressed locally in skeletal muscle (Fig. S4e, f), which is particularly interesting given recent observations that poly-GA is commonly found there in human *C9orf72* ALS cases [9]. Thus, selective loss of motoneurons and neuromuscular denervation together with potential cell-autonomous effects in the muscle are likely the predominant drivers of progressive muscle wasting in GA-Nes mice.

### TDP-43 and other disease-linked RNA-binding proteins form nuclear inclusions in GA-Nes mice

**Fig. 4:**
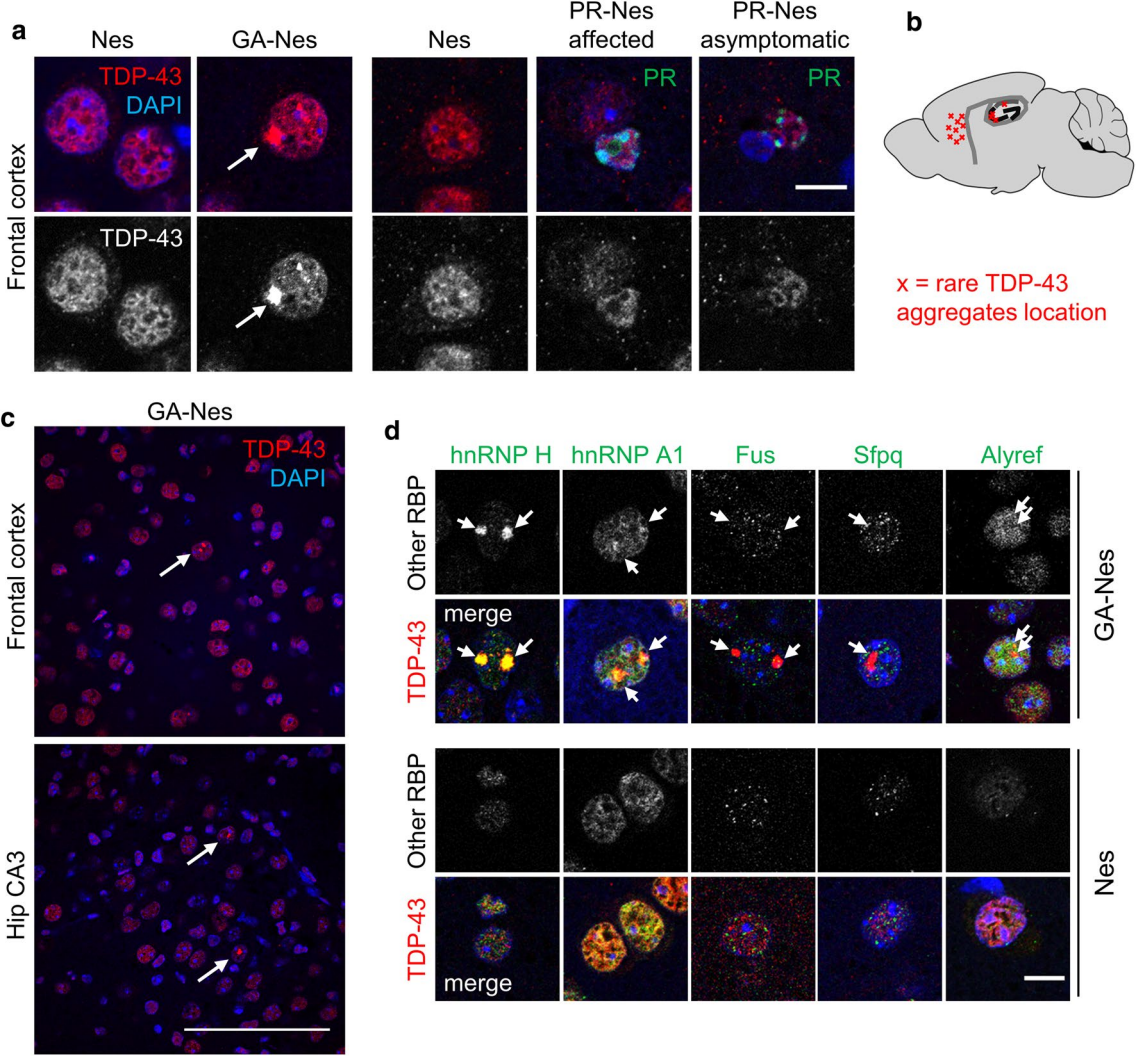
TDP-43 and other disease-linked RNA-binding proteins form nuclear inclusions in GA-Nes mice. **a** Neuronal nuclear inclusions of TDP-43 (arrow) are detected by immunofluorescence in hippocampus and frontotemporal cortex in GA-Nes end-stage mice, but not Nes littermate controls. No TDP-43 inclusions are detected in affected or asymptomatic PR-Nes mice, or a 68-week Nes control, even in cells with abundant poly-PR (green) inclusions. Scale bar = 10 µm. **b** Schematic drawing of the distribution of the rare nuclear TDP-43 aggregates in GA-Nes mice. Aggregates were found in the lateral frontal cortex (at least 100 µm from the midline) and hippocampal CA regions marked with red *x*. **c** Regional overview of TDP-43 nuclear aggregates (arrows) in the frontal cortex and hippocampus CA3 region of end-stage GA-Nes mice, scale bar = 100 µm. **d** Nuclear TDP-43 aggregates (arrows) in end-stage GA-Nes mice colocalize with hnRNP H, and weakly with hnRNP A1, but not with Fus, Sfpq or Alyref. No nuclear aggregates of any marker are seen in age-matched Nes mice. Scale bar = 10 µm

To elucidate the elusive link between DPR and TDP-43 pathology, we characterized TDP-43 localization in GA-Nes and PR-Nes mice in detail using a panel of antibodies (Fig S5a). In the cortex of control mice, TDP-43 staining with an N-terminal antibody showed the typical nuclear network-like pattern (Fig. 4a). TDP-43 expression seemed more diffuse in GA-Nes mice and to a lesser extent also in PR-Nes mice, but we did not observe nuclear clearance or cytoplasmic TDP-43 aggregates in GA-Nes and PR-Nes lines and did not observe additional cleavage products in the RIPA-insoluble fraction of GA-Nes mice by Western blot (Fig S5b). However, we noticed occasional nuclear TDP-43 aggregates (~ 1% of neurons) specifically in the lateral frontal cortex and hippocampal CA regions of GA-Nes mice (Fig. 4a–c). In contrast, even in neurons with numerous nuclear poly-PR aggregates, no nuclear or cytoplasmic inclusions of TDP-43 were observed in asymptomatic or affected PR-Nes mice (Fig. 4a). We detected these nuclear TDP-43 inclusions in GA-Nes mice with several N- and C-terminal antibodies, and comparison of C-terminal antibodies specific for non-phospho- or phospho-TDP-43 showed that the nuclear aggregates are not phosphorylated at serine 403/404 or serine 409/410 (Fig. S5a).

To further characterize the TDP-43 nuclear pathology in GA-Nes mice, we investigated co-aggregation of other RNA-binding proteins implicated in disease, as well as proteins involved in general RNA trafficking and splicing functions. Unexpectedly, immunostaining revealed strong colocalization of nuclear TDP-43 aggregates with hnRNP H, which has been linked to RNA toxicity in *C9orf72* cases, and weak colocalization with hnRNP A1 previously implicated in ALS/FTD [30, 33] (Fig. 4d). Absence of colocalization with the RNA-binding proteins FUS, SFPQ or ALYREF demonstrates the specificity of the interaction (Fig. 4d). Thus, only poly-GA promotes sparse nuclear aggregation of TDP-43 and other hnRNPs implicated in *C9orf72* disease in a region-specific manner.

### Poly-GA downregulates a network of synaptic functional genes and triggers pro-inflammatory responses

To allow unbiased investigation of disease-related CNS pathomechanisms in our mouse models, we performed RNA sequencing of bulk tissue. For GA-Nes mice, we compared brainstem, cortex, hippocampus and spinal cord at end-stage and at 3 weeks (for the brain regions). For PR-Nes, we analyzed the cortex and hippocampus of 4-week affected and 68-week-old asymptomatic mice. The full expression data are listed in Table S1.

**Fig. 5:**
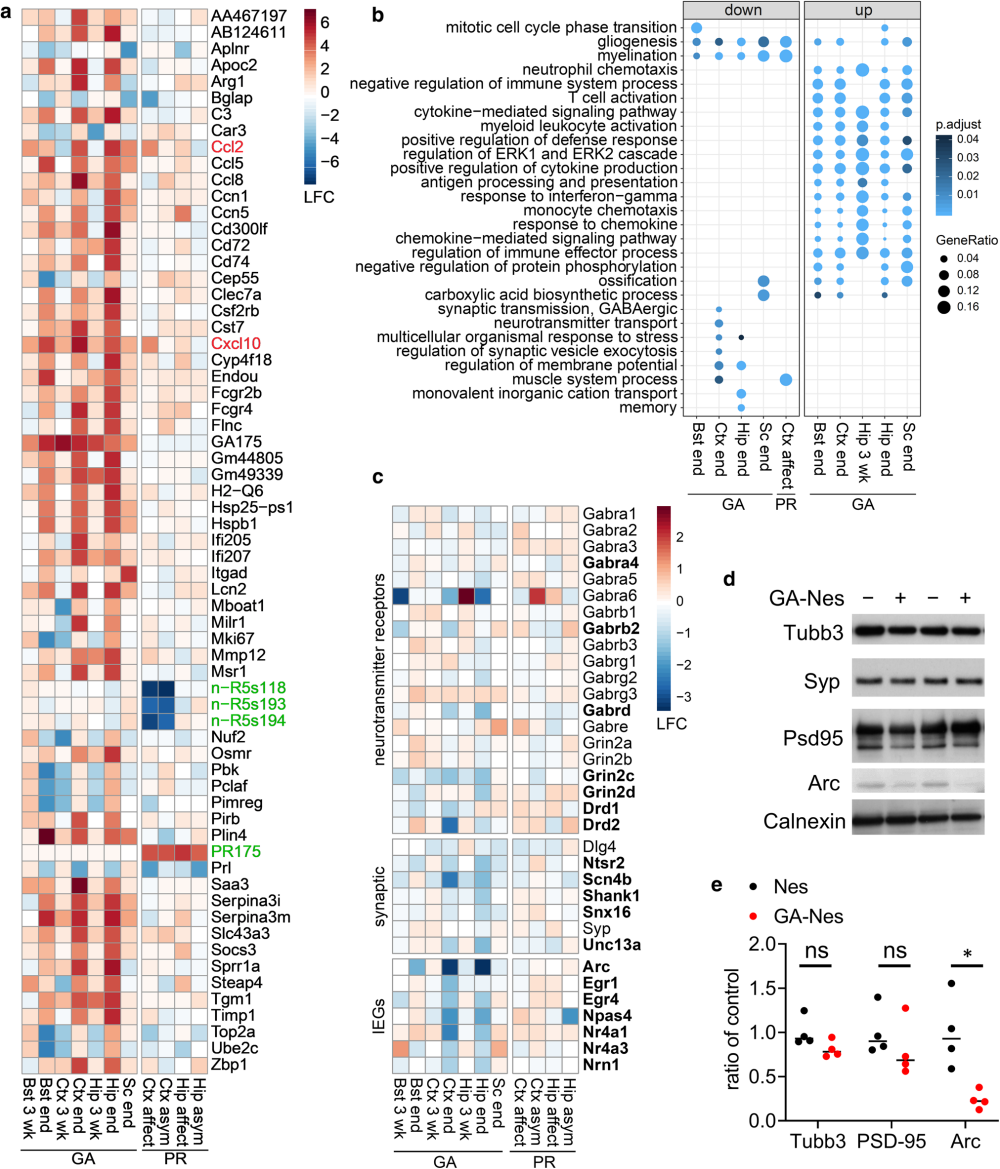
Poly-GA reduces many genes important for synaptic function and triggers inflammation. **a** Significantly regulated genes in GA-Nes and PR-Nes with absolute log_2_-fold expression changes larger than 4.5 in at least one condition. Significant differences are in PR-Nes mice are noted in green, and those overlapping between GA-Nes and PR-Nes are noted in red. The remaining genes are only differentially expressed in GA-Nes, predominantly at end-stage. For the full list of differentially expressed genes, see Tables S1 and S2a/b. **b** Gene ontology analysis of differentially expressed genes (compare Tables S2 and S3) for the indicated regions and time points. The top 5 non-overlapping terms with more than 5 genes are shown for each group (groups that do not meet these criteria are not shown). The largest cluster among downregulated genes is related to synaptic function in GA-Nes, and among upregulated genes is related to immune response in GA-Nes. Genes per group from left to right: 193, 226, 264, 50, 58, 372, 585, 42, 991, 90. The dot size and color represent the fraction of the differentially expressed genes in each category and adjusted *p* values, respectively. **c** Heatmap of genes in the synaptic terms (including membrane potential and memory) identified in **b** are most consistently reduced in GA-Nes Ctx and Hip at end-stage, but not at 3 weeks or in PR-Nes. Bold genes are differentially expressed in at least one condition (adjusted *p* < 0.05). **d** Representative western blots from the neocortex show reduced Arc levels, but no change in selected neuronal proteins in end-stage GA-Nes (+) vs. Nes controls (−). Calnexin is used as a loading control. **e** Protein quantification of neuronal markers in **d**, β-tubulin III (Tubb3) and PSD-95 (Dlg4) shows no significant change in protein levels in end-stage GA-Nes mice [Tubb3: Mann–Whitney *t* test *U* = 2, ns *p* = 0.114; PDS-95 *t* test *t*(6) = 0.939, ns *p* = 0.384], while functional marker Arc is significantly reduced [Welch’s *t* test *t*(3.39) = 3.58, *p = 0.03]. *n* = 4/group

Principal component analysis showed strong separation of the genotypes in different brain regions in GA-Nes end-stage and affected PR-Nes mice, but not in GA-Nes mice at 3 weeks of age, which further excludes developmental effects due to poly-GA expression (Fig. S6a). Correlation analysis revealed overall similar gene expression changes in the different brain regions of GA-Nes mice particularly at the end-stage time point (Fig. S6b). We analyzed differential gene expression for all regions and time points with DESeq2. In total 2351 genes were significantly up- or down-regulated in GA-Nes mice, compared with only 123 genes in PR-Nes mice and 53 in both using our cut-off criteria (adjusted *p* < 0.05, see Table S2). The genes showing the strongest differential expression in GA-Nes and PR-Nes are shown in Fig. 5a. We detected the strongest changes in the GA-Nes hippocampus, in line with the early pronounced microglial activation and neuron loss visible in this area (compare Fig. 2f). The strongest change in PR-Nes mice is the reduced expression of three 5S rRNA transcripts in the cortex of both affected and asymptomatic mice, which corroborates the known effects of poly-PR in inhibiting protein translation [21]. Among the top differentially expressed genes, only Ccl2 and Cxcl10 were significantly different in both GA-Nes and PR-Nes mice. Gene expression changes in asymptomatic PR-Nes mice are minimal and revealed no obvious mechanistic basis for the transgene repression in asymptomatic compared to affected mice (Table S1).

Among down-regulated transcripts, gene ontology (GO) analysis identified enrichment of genes encoding synaptic proteins (e.g., neurotransmitter receptor subunits and proteins involved in vesicle release) as well as immediate early genes associated with learning and memory in GA-Nes cortex and hippocampus at end-stage, but not in PR-Nes tissues (Fig. 5b, c). Comparison of significant hits (bold), related subunits of GABA, NMDA and dopamine receptors and other key synaptic genes suggests altered synaptic function in GA-Nes mice may result in reduced activation of immediate early genes such as Arc, which is critical for learning and memory [42] (Fig. 5c). Analysis of protein levels in neocortex by western blot showed that the immediate early gene Arc is indeed significantly reduced in the end-stage cortex of GA-Nes mice, but levels of key neuronal proteins β-tubulin-III and PSD-95 were not reduced (Fig. 5d, e). These findings are consistent with the absence of neuron loss in this region by the time of euthanasia (see Fig. 2i). Myelination-associated genes (Klk6, Mal, Pllp, Plp1, Fa2h, Hes5, Aspa) were also downregulated in GA-Nes and PR-Nes mice, likely due to DPR expression in a subset of oligodendrocytes (see Fig. S1f), which has so far not been reported in *C9orf72* patients.

Overall, up-regulated transcripts in GA-Nes mice were strongly enriched for immune responses, including cytokine- and chemokine-mediated signaling and interferon-inducible genes such as Ifi205/207 and chemokines in all regions in end-stage GA-Nes but not (or much less) PR-Nes mice (Fig. 5a, Table S3). Thus, poly-GA expression induces pronounced neuroinflammation and may also impair synaptic function.

### Poly-GA triggers microglial interferon responses enriched in *C9orf72* ALS

**Fig. 6:**
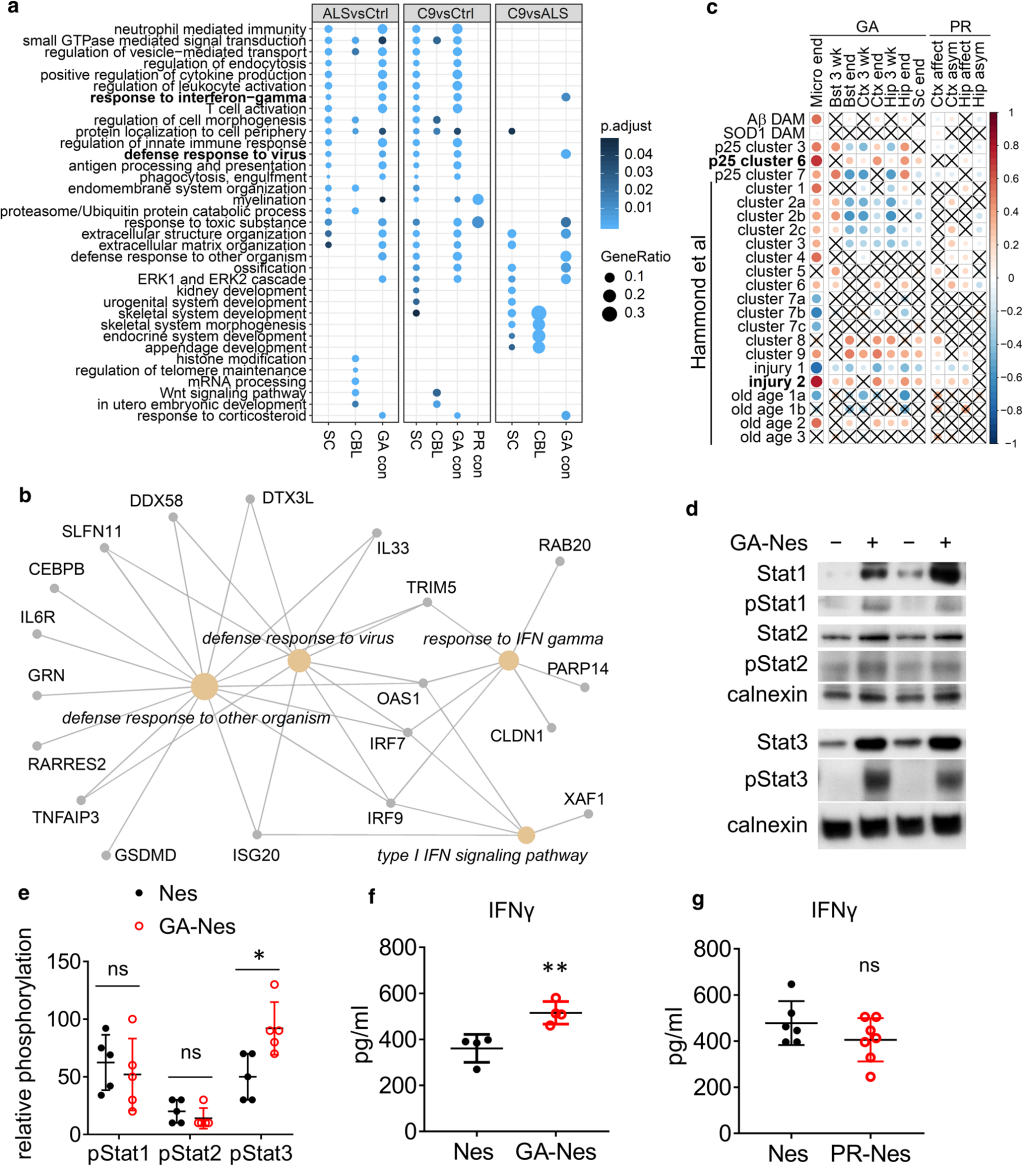
Poly-GA, but not poly-PR, triggers microglial pro-inflammatory responses characteristic of disease-associated microglia, aging, and interferon that are similar to *C9orf72* ALS patients. **a** Gene ontology analysis of genes differentially expressed between ALS cases without (“ALS”) or with *C9orf72* (“C9”) mutation compared to controls (“Ctrl”). Terms with > 5 genes that are concordantly regulated in GA-Nes (GA con) or affected PR-Nes (PR con) mice are also analyzed. Concordantly regulated genes were defined by comparing averaged significant up/down-regulation from all analyzed regions in GA-Nes or affected PR-Nes mice and ALS patients. Number of genes in each group from left to right: 5810, 4742, 660, 5351, 1279, 689, 45, 999, 33, 111. SC spinal cord, CBL cerebellum. Full data is available in Table S4 and S5. **b** Network of the immune-related genes concordantly regulated in GA-Nes mice and *C9orf72*-positive vs *C9orf72*-negative ALS patients. **c** Correlation analysis of gene expression in isolated microglia from GA-Nes mice or whole tissue from different brain regions in GA-Nes- and PR-Nes-affected (affect) and asymptomatic (asym) mice with differentially expressed genes in microglial subpopulations identified by single-cell RNAseq in Aβ and SOD1 mouse models [26], p25 neurodegeneration mice [40] and wildtype mice during development (cluster 1–9), injury and aging [19]. Pearson’s correlation coefficient indicated by color and circle size. Non-significant correlations are crossed out. Size of the published genes sets from top to bottom: 1482, 4589, 730, 955, 271, 278, 332, 483, 295, 678, 452, 118, 158, 40, 83, 52, 66, 183, 192, 188, 22, 12, 130, 36. Strongest positive correlation with GA-Nes microglia is found in interferon-response clusters injury 2 and p25 cluster 6. **d** Immunoblots from whole neocortex of total and phosphorylated Stat effector proteins of interferon signaling in end-stage GA-Nes (+) and age-matched Nes controls (−). Calnexin is used as a loading control. **e** Relative phosphorylation levels normalized to total protein levels increase in end-stage GA-Nes only for Stat3 compared to age-matched Nes controls [Two-way ANOVA (effector × genotype) *F*(2,24) = 3.88, *p* = 0.035 *η*^2^ = 0.323. Sidak post hoc pStat1 Nes (62.4 ± 24.0) vs GA-Nes (52 ± 31.1) ns *p* = 0.858, pStat2 Nes (20 ± 10) vs GA-Nes (14 ± 8.9) ns *p* = 0.998*,* pStat3 Nes (38.6 ± 17.3) vs GA-Nes (82.6 ± 24.9) * *p* = 0.017], *n* = 5/group. **f** ELISA for IFNγ in whole neocortex homogenate is significantly increased in end-stage GA-Nes mice. [Mann–Whitney Nes (361 ± 60.7) vs GA-Nes (515 ± 49.3) **p = 0.029, 97% CI 62.3–310] *n* = 4/group. **g** IFNγ in whole neocortex homogenate is not significantly changed in affected PR-Nes mice compared to Nes littermate controls. [*t*(10) = 1.661, Nes (478 ± 94.9) vs PR-Nes affected (389 ± 91), *p* = 0.128 *η*^2^ = 0.216] *n* = 6/group. Power to detect *η*^2^ > 0.5 = 83.7%

To examine the relevance of these findings to human disease, we correlated gene expression changes in GA-Nes mice with large public transcriptome data of spinal cord, motor cortex and cerebellum from ALS patients (TargetALS). All regions showed clear expression changes in ALS cases (see full expression data in Table S4). Surprisingly, gene expression changes in all GA-Nes regions correlated better with spinal cord than motor cortex from both *C9orf72* and non-*C9orf72* ALS patients, and inversely correlated with cerebellum and frontal cortex (Fig. S6c). In contrast, PR-Nes mice showed only weak correlations with human ALS tissue. Comparing gene expression in *C9orf72*-positive and *C9orf72*-negative cases (C9vsALS), confirmed the upregulation of homeobox genes in the cerebellum (e.g., HOXA5 [15]) and showed altered expression of genes related to the extracellular matrix in the spinal cord of *C9orf72* cases (Table S5 and Fig. 6a). GO analysis of genes concordantly regulated in GA-Nes mice and the C9vsALS patient data also showed enrichment of interferon signaling (e.g., IRF7 and IRF9) and defense response genes (e.g., GRN), while no GO terms were concordantly enriched in both PR-Nes and C9vsALS using our criteria (Fig. 6a). The network of these immune-related genes concordantly upregulated in GA-Nes mice and C9vsALS brains is shown in Fig. 6b.

To further verify whether the pro-inflammatory responses were driven by microglia, we performed RNA sequencing on isolated microglia from end-stage GA-Nes brain. Transgene-specific sequence tags (GA175) further confirm that poly-GA is not expressed in microglia (Fig. S6d). The top differentially expressed genes in GA-Nes microglia include many interferon-stimulated genes (e.g., MX1, Isg15, Oasl1/2 and various Ifi genes) and complement factors (C3 and C4b) (Fig. S6d). Therefore, we further compared our data to different microglial subtypes recently identified by single-cell transcriptome studies of microglia under physiological and pathological conditions [19, 40]. The strongest positive correlations were with cluster 6 from a p25 neurodegeneration model [40] and in a lysolecithin demyelination model (injury 2) [19], two microglia subpopulations with strong induction of interferon-response genes (Fig. 6c). Comparing this with the bulk tissue sequencing, these signatures also correlate moderately in GA-Nes end-stage tissues, but weakly or not at all in PR-Nes (Fig. 6c). Comparing only the signature genes of several published microglia populations shows the greatest overlap with interferon-response microglia (IRM) in a mouse model of Alzheimer's disease [56] (Fig. S6e). Together, these data indicate that poly-GA expression induces a proinflammatory microglial response dominated by interferon signaling.

We confirmed the interferon response in end-stage GA-Nes mice by immunoblotting for the STAT proteins that act as intermediate effectors of interferon signaling. The upregulation of total STAT1 and STAT3 suggests strong microglial proliferation, and the upregulation of phosphorylated STAT3 relative to total in GA-Nes cortex (Fig. 6d, e) is consistent with an interferon response. *Stat3* gene expression was also significantly elevated at end-stage in hippocampus and cortex (Table S2). Measuring IFNγ directly by ELISA showed that IFNγ was significantly elevated in end-stage GA-Nes but not affected PR-Nes mice (Fig. 6f, g). Taken together, our data indicate that poly-GA inclusions trigger interferon responses in microglia that are also enriched in human *C9orf72* ALS.

## Discussion

Using novel mouse models for conditional high-level expression of key DPRs, we show that poly-GA triggers rapidly progressive neuroinflammation, muscle denervation, and selective neuronal loss in all mice. A congenic poly-PR line with the same repeat length and similar mRNA levels produced far lower DPR aggregation resulting in ataxia and seizures requiring early euthanasia in a subset of animals. However, 60% of animals were able to suppress poly-PR mRNA expression to non-toxic levels, which importantly were still higher than the frequency of poly-PR reported in human tissues [38, 58]. In addition, we found no clear ALS/FTD-related pathology in either affected or asymptomatic PR-Nes mice. Thus, our comparative study highlights the role of poly-GA in *C9orf72* ALS/FTD by triggering neuron loss and neuroinflammation, and our GA-Nes mouse model will allow rapid testing of poly-GA-directed therapy in the future.

### Differential aggregation and toxicity of poly-GA and poly-PR in vivo

Poly-PR is highly toxic in mammalian cells, yeast and drosophila by interfering with stress granule formation, nucleocytoplasmic transport, heterochromatin organization, nucleolar dynamics and protein translation by affecting the liquid-liquid phase separation of other low-complexity proteins [2, 3, 21, 24, 31, 32, 44, 68]. While poly-PR localized to heterochromatin and the nucleolus in both affected and asymptomatic PR-Nes mice as in other models, we observed no neuron loss in PR-Nes animals. In addition, asymptomatic PR-Nes mice seem to escape lethality by suppressing the expression of the synthetic transgene mRNA. Thus, nuclear poly-PR toxicity is dose dependent, consistent with another recent report [20]. In addition, post-transcriptional suppression mechanisms likely exist in vivo, because most neurons outside the hippocampus in affected PR-Nes mice have no detectable poly-PR aggregation. Indeed, we find reduced expression of 5S rRNA in the cortex but not hippocampus in PR-Nes mice that may contribute to reduced poly-PR protein levels in the cortex, and is consistent with poly-GR/PR-mediated inhibition of translation [21, 25, 35, 66].

In contrast, poly-GA shows modest toxicity in rodent primary neurons by gradually sequestering the proteasome and other proteins [18, 41]. We confirmed sequestration of the proteasome in our GA-Nes mice and occasional nuclear aggregation of TDP-43 and other low-complexity RNA-binding proteins suggests poly-GA impairs proteostasis in vivo, as we showed recently in vitro [18, 29]. Surprisingly, using identical expression systems, poly-GA toxicity and aggregation was more severe in the mouse central nervous system and resulted in a more ALS-like phenotype than poly-PR expression. Proteasomal impairment, indicated by partial sequestration of Psmc4 in GA-Nes, may contribute to the strong accumulation of poly-GA compared to poly-PR despite similar mRNA levels in affected PR-Nes mice, consistent with data from cellular models [41]. Overall, direct comparison of our mouse lines indicates a combination of post-transcriptional mechanisms could explain the predominant aggregation of poly-GA compared to poly-PR seen in *C9orf72* patients without invoking RAN translation-mediated effects.

### Widespread poly-GA but not poly-PR expression causes disease-relevant phenotypes

In our models, poly-GA led to selective neuron loss in the hippocampus and spinal cord as well as muscle denervation resembling ALS. In contrast, poly-PR expressed at the same mRNA level led to ataxia and seizures without obvious neuron loss or inflammation in the brain or spinal cord. While these findings confirm toxicity of high nuclear poly-PR levels, they do not replicate ALS phenotypes well. However, since national animal welfare regulations required us to euthanize mice after a single stage II seizure (see “Materials and methods”), we cannot exclude that repeated seizures would lead to overt neuron loss in these mice. More ALS-like toxicity of poly-PR has been observed in AAV-based expression models or with homozygous transgenic expression that drives higher poly-PR levels compared to our single-copy transgenic line, leading to weight loss and early lethality in a subset or all mice, respectively [20, 68]. Both of these poly-PR mouse models also showed ataxia-like phenotypes due to Purkinje cell loss, which was not detected in our PR-Nes model (data not shown), and one line showed loss of lower motor neurons and inflammation within the spinal cord [20]. In contrast, in our PR-Nes line, poly-PR inclusions are enriched in the hippocampus, a common focal point of epilepsy and the area where poly-PR aggregates have been reported to be most abundant in human *C9orf72* FTD [38, 58]. However, while seizures and epilepsy have been reported in *C9orf72* patients, they are not a common feature [5]. The different phenotypes of low and high-expressing PR-Nes mice further support dose-dependent toxicity of poly-PR. However, even asymptomatic PR-Nes mice have more abundant poly-PR inclusions than *C9orf72* patients [38, 58]. While the scarcity of poly-PR inclusions in patients has been widely attributed to its high toxicity in vitro [62, 68], the modest transcriptional changes in PR-Nes mice despite Nestin-Cre-driven expression of the poly-PR transgene throughout the CNS under the control of the strong CAG promoter do not support dominant effects of barely detectable poly-PR aggregates in patients. Nevertheless, we cannot rule out that the predominantly cytoplasmic localization of poly-PR in patients [16, 58] and the longer repeat length could still cause more dramatic effects in patients.

Overall, our poly-GA mice model several key aspects of *C9orf72* ALS well, including selective loss of motor neurons in the spinal cord, muscle denervation, and an interferon-response microglial signature concordant with *C9orf72* ALS. In addition, the sparse nuclear TDP-43 inclusions only in the forebrain are reminiscent of *C9orf72* FTD. Together these data provide in vivo evidence for selective vulnerability towards poly-GA that may explain the disconnect between *C9orf72*-specific pathology and neurodegeneration in patients. However, it is necessary to understand the three limitations of this model to maximize its usefulness. First, our mice show modest poly-GA expression in some cells in kidney, pancreas and GI tract, which has not been reported in *C9orf72* patients [1]. While histopathological analysis nevertheless revealed overall normal organ morphology and poly-GA expression in the neurons of the myenteric plexus of GA-Nes mice does not seem to impair peristalsis (based on observation of the intact GI tract), we cannot rule out more subtle functional deficits. Investigating whether DPR inclusions occur in the enteric nervous system of *C9orf72* patients could be worthwhile because cachexia is a common but poorly understood feature of sporadic and *C9orf72* ALS that often precedes the diagnosis by many years [52]. Second, although the distribution of DPR inclusions is predominantly neuronal a subset of oligodendrocytes and astrocytes do express transgene RNA. Third, GFP-only control littermates would have been ideal to rule out any effects of GFP alone, but this was not possible with our construct design. However, we are not aware that any of the large number of published mouse lines expressing high levels of GFP in the brain have shown phenotypes similar to our GA-Nes mice. Moreover, the largely non-overlapping phenotypes of our GA-Nes and PR-Nes mice argue against non-specific off-target effects due to the widespread expression of DPR proteins or GFP using the Nestin-Cre driver.

An important aspect of our novel poly-GA mouse model is that all mice reached the predefined endpoints according to national animal welfare regulations before 7 weeks, while abundant data from the neuromuscular junctions, motor neurons, and transcript analysis indicate no developmental defects. This consistent and rapid progression with motoneuron loss, denervation and *C9orf72*-enriched transcriptional changes are unique features of this *C9orf72* model and offer an advantage for analyzing poly-GA-targeted therapy in vivo. As in other ALS models and patients, denervation is more pronounced than motor neuron loss arguing for the contribution of dying-back mechanisms due to synaptic toxicity [10]. In addition, poly-GA expression in muscle tissue could contribute to denervation and weakness in GA-Nes mice, and recent studies have uncovered that poly-GA is expressed in the skeletal muscle of *C9orf72* patients [9]. Crossing our conditional line with other Cre drivers will allow us to dissect the role of peripheral poly-GA expression in the future.

### TDP-43 and other disease-linked RNA-binding proteins form nuclear inclusions in GA-Nes mice

In addition to DPR inclusions, TDP-43 inclusions are characteristic of sporadic and *C9orf72*-related and other forms of ALS/FTD and correlate far better with neurodegeneration [37]. However, it is still debated how TDP-43 pathology is triggered by the *C9orf72* mutation. The lack of spatial correlation of DPR expression, RNA foci and *C9orf72* expression with TDP-43 aggregates in patients suggests that selective vulnerability is critical. This is supported by the striking finding that nuclear TDP-43 aggregates selectively in a subset of neurons in the hippocampus and frontal cortex of GA-Nes mice, despite abundant poly-GA aggregation throughout the brain. Study of the pathomechanism in mice is complicated by differences between mouse and human molecular biology, in particular the processing by caspase 4 that generates the aggregation-prone C-terminal fragments found in cytoplasmic inclusions [64]. Rare cytoplasmic inclusions of TDP-43 have been reported in some (G_4_C_2_)_*n*_ models and viral poly-GA expressing mouse models, but most inclusions in these mice are nuclear [6, 23, 34, 66]. In our congenic mouse models, only poly-GA induced regional intranuclear aggregates of TDP-43 that were not phosphorylated at the disease-specific sites S409/410 or S403/404. We did not observe disease-like proteolytic processing of TDP-43 or cytoplasmic TDP-43 inclusions. Nevertheless, the nuclear TDP-43 aggregates partially colocalize with other RNA-binding proteins hnRNP H identified as an interactor of (G_4_C_2_)_*n*_ RNA, and hnRNP A1 also implicated in ALS/FTD [8, 30, 33]. We now show that poly-GA alone is sufficient to induce aggregation of hnRNP H, in the absence of (G_4_C_2_)_*n*_ RNA. Overall, although these nuclear inclusions are rare at the young age of our mice, our data suggest that poly-GA impairs proteostasis of disease-linked RNA-binding proteins, perhaps mediated by proteasome sequestration [18, 29]. Together with other insults such as defective RNA transport and caspase-mediated cleavage, this may ultimately lead to their cytoplasmic aggregation in patients.

### Poly-GA triggers pro-inflammatory interferon responses similar to human ALS spinal cord

GA-Nes mice show strong activation of microglia throughout the CNS, but particularly in the hippocampus where neurons are lost at an early age. We used RNAseq to elucidate the inflammatory response and conducted a time-course study in multiple CNS regions in GA-Nes and PR-Nes mice. The gene expression profiles are dominated by chemokines and other inflammatory changes in GA-Nes mice with the strongest effects in the degenerating hippocampus. In addition, synaptic genes and several immediate early genes such as Arc, which is crucial for memory formation [42], are downregulated. Together with our observation that cortical neurons are not lost by this age in GA-Nes mice, these data suggest that poly-GA impairs synaptic function, which may contribute to *C9orf72* pathogenesis prior to overt neuron loss [54].

Comparing differential gene expression in GA-Nes mice and human ALS cases shows a strong overlap with an inflammatory signature common to both *C9orf72* and sporadic ALS. Comparing gene expression in GA-Nes mice and *C9orf72*-specific changes in human ALS spinal cord highlights the role of enhanced interferon signaling. Interestingly, IFNα levels are higher in the CSF of *C9orf72* ALS compared to sporadic ALS [51]. Transcriptome analysis in cortex samples from ALS cases identified a subgroup of patients with high microglia/interferon response, without enrichment in *C9orf72* cases, but the spinal cord was not analyzed in that study [60]. Comparing gene expression of isolated GA-Nes microglia to recently published microglia expression signatures from single cell RNAseq experiments [19, 26, 40, 56] highlights the role of pro-inflammatory microglia subpopulations characterized by a strong interferon response. Indeed, many of the differentially expressed genes are known to be interferon-inducible and we show that STAT signaling is enhanced in GA-Nes mice. In addition, we confirmed that IFNγ was increased in GA-Nes but not PR-Nes mice. Moreover, CHIT1, a sensitive CSF biomarker for ALS [50, 59] is also strongly induced by interferon [39] suggesting a sterile interferon response is highly relevant in human ALS. A causal role of interferon in neurodegeneration is supported by inherited interferonopathies such as Aicardi–Goutieres syndrome, associated with cerebral atrophy and psychomotor retardation in children [17]. We show for the first time that poly-GA is sufficient to induce strong interferon responses in vivo, which is seen in the spinal cord of sporadic ALS and enriched in *C9orf72* patients. Future experiments to investigate whether inhibiting this pro-inflammatory response will extend the lifespan of GA-Nes animals would help determine whether this may be a therapeutic approach for *C9orf72* ALS.

## Conclusion

Taken together, our GA-Nes mice reproduce key features of *C9orf72* ALS/FTD far better than the congenic PR-Nes mice. In addition, we identify a *C9orf72*-enriched inflammatory signature triggered by poly-GA but not poly-PR in mice that supports a dominant role of poly-GA in *C9orf72* ALS/FTD pathogenesis. In contrast, the low abundance of poly-PR aggregates in patients and post-transcriptional reduction of poly-PR in most mouse tissues (perhaps through down-regulation of 5S rRNA) suggests that neurons in humans may also be capable of suppressing poly-PR aggregation and emphasizes the link to ribosome-mediated toxicity. Since poly-GA triggers region-specific neuron loss and down-regulation of synaptic genes essential for memory and induces an inflammatory milieu similar to ALS spinal cord, we propose poly-GA and subsequent inflammatory responses are important targets for *C9orf72* therapeutics. Despite some limitations, our novel model for *C9orf72* ALS/FTD with widespread high-level poly-GA expression, selective vulnerability in spinal cord motor neurons, early onset and rapid progression will be useful for the initial preclinical testing of therapies to block poly-GA toxicity and associated pro-inflammatory interferon responses in microglia.

## Electronic supplementary material

Below is the link to the electronic supplementary material.

Supplementary file1 (XLSX 29027 kb)

Supplementary file2 (PDF 1907 kb)

Supplementary file3 (XLSX 573 kb)

Supplementary file4 (XLSX 35995 kb)

Supplementary file5 (XLSX 766 kb)

Supplementary file6 (MP4 28463 kb)

Supplementary file7 (MP4 53260 kb)

Supplementary file8 (MP4 19826 kb)

Supplementary file9 (MP4 48686 kb)

Supplementary file10 (PDF 2946 kb)
