# Supplementary material for: Congenic expression of poly-GA but not poly-PR in mice triggers selective neuron loss and interferon responses found in C9orf72 ALS
Source: Acta Neuropathol. 2020 Jun 19;140(2):121–42. doi: 10.1007/s00401-020-02176-0 (PMC7360660; doi:10.1007/s00401-020-02176-0)

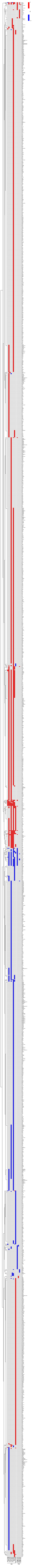

Supplement: Supplementary file 2 — Supplementary file2 (PDF 1907 kb) [file 401_2020_2176_MOESM2_ESM.pdf]
