# Supplementary material for: Congenic expression of poly-GA but not poly-PR in mice triggers selective neuron loss and interferon responses found in C9orf72 ALS
Source: Acta Neuropathol. 2020 Jun 19;140(2):121–42. doi: 10.1007/s00401-020-02176-0 (PMC7360660; doi:10.1007/s00401-020-02176-0)
Supplement: Supplementary file 10 — Supplementary file10 (PDF 2946 kb) [file 401_2020_2176_MOESM10_ESM.pdf]

Electronic supplemental materials

Congenic expression of poly-GA but not poly-PR in mice triggers selective neuron loss and interferon responses found in *C9orf72* ALS

Katherine D. LaClair[1,#], Qihui Zhou[1,2,#], Meike Michaelsen[1], Benedikt Wefers[1], Monika S. Brill[2,3], Aleksandar Janjic[4], Birgit Rathkolb[5,6,7], Daniel Farny[1], Mikolaj Cygan[1], Martin Hrabe de Angelis[5,7,8], Wolfgang Wurst[1,2], Manuela Neumann[9,10], Wolfgang Enard[4], Thomas Misgeld[1,2,3], Thomas Arzberger[1,2,11,12], Dieter Edbauer[1,2,13,*]

1 German Center for Neurodegenerative Diseases (DZNE), Munich, 81377 Munich, Germany
2 Munich Cluster for Systems Neurology (SyNergy), 80336 Munich, Germany
3 Institute of Neuronal Cell Biology, Technische Universität München, 80802 Munich, Germany
4 Department Biology II, Anthropology and Human Genomics, Ludwig-Maximilians-University, 82152, Martinsried, Germany.
5 German Mouse Clinic, Institute for Experimental Genetics, Helmholtz Zentrum München, German Research Center for Environmental Health, Neuherberg, Germany
6 Institute of Molecular Animal Breeding and Biotechnology, Gene Center, Ludwig-Maximilians-University Munich, 81377 Munich, Germany
7 German Center for Diabetes Research (DZD), Ingolstädter Landstr. 1, 85764 Neuherberg, Germany
8 Chair of Experimental Genetics, School of Life Science Weihenstephan, Technische Universität München, Alte Akademie 8, 85354 Freising, Germany
9 German Center for Neurodegenerative Diseases (DZNE), Tübingen, Germany
10 Department of Neuropathology, University of Tübingen, Tübingen, Germany
11 Center for Neuropathology and Prion Research, Ludwig-Maximilians-University Munich, 81377 Munich, Germany
12 Department of Psychiatry and Psychotherapy, University Hospital, LMU Munich, 80336 Munich, Germany
13 Ludwig-Maximilians-University Munich, Graduate School of Systemic neurosciences (GSN), 81377 Munich, Germany
# These authors contributed equally.
* Correspondence should be addressed to dieter.edbauer@dzne.de

Table of contents

- **Supplemental Figure S1-S6**
- **Legends to supplemental Videos 1-4**
- **Legends to supplemental Tables S1-S6**
- **Supplemental References**

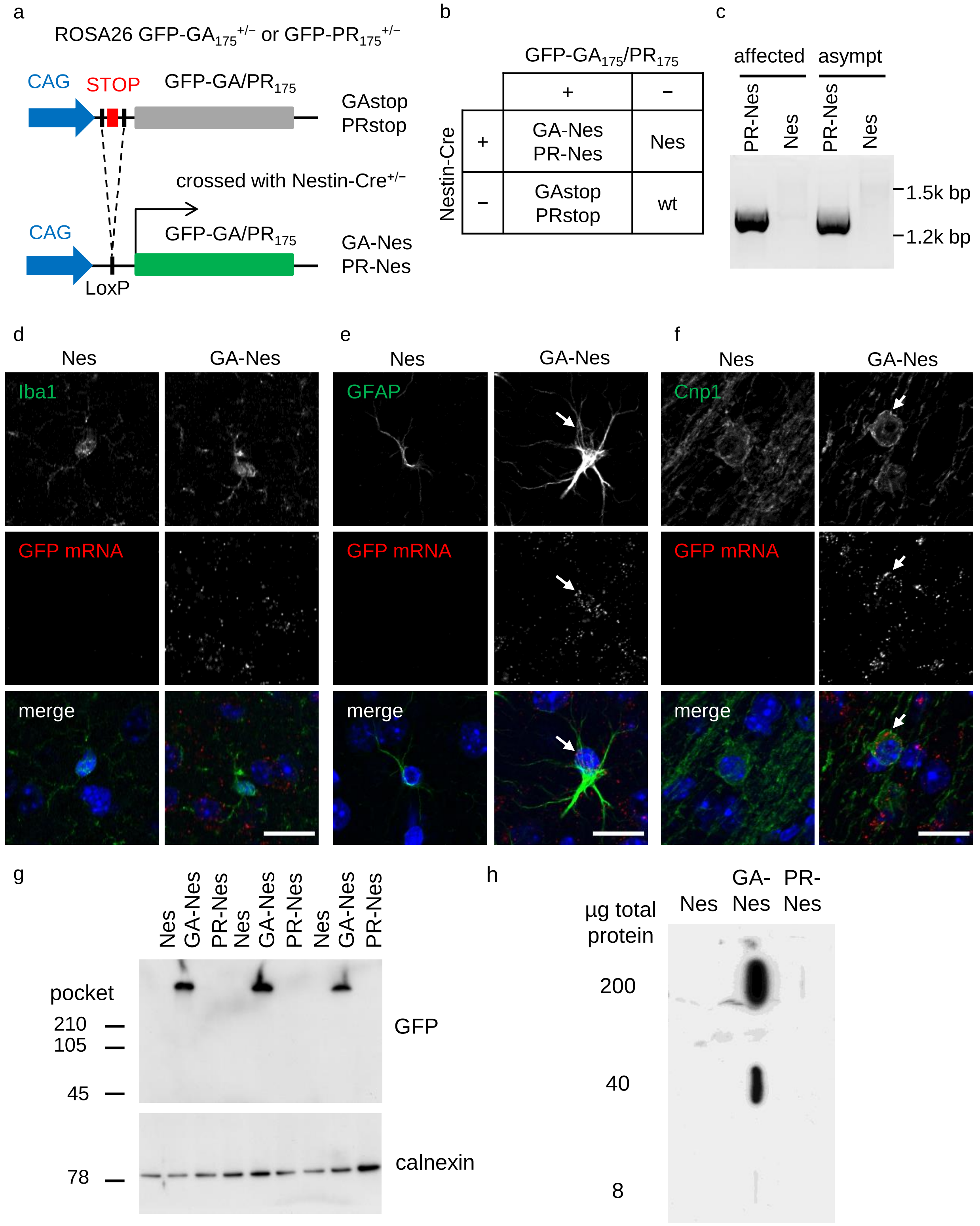

Fig. S1. Generation of conditional DPR-expressing mice and pattern of transgene expression

a) Graphic representation of conditional poly-GA and poly-PR expression and mouse breeding strategy, described in Methods. Rosa26 integration results in congenic single-copy lines. The strong CAG promoter drives DPR expression upon Cre-recombination.

b) Naming of genotypes generated by breeding strategy in a).
c) PCR of the poly-PR transgene shows equally sized bands in affected and asymptomatic PR-Nes mice and no truncation products.
d) FISH of GFP mRNA combined with Iba1 immunofluorescence shows no poly-GA expression in microglia.
e) FISH of GFP mRNA combined with GFAP immunofluorescence shows poly-GA expression in a subset of astrocytes.
f) FISH of GFP mRNA combined with Cnp1 immunofluorescence shows poly-GA expression in a subset of oligodendrocytes.
g) Comparative immunoblot using a GFP antibody in Nes, end stage GA-Nes and affected PR-Nes mice shows strong accumulation of poly-GA in the pocket of the gel, while no poly-PR aggregates or soluble protein can be detected. Calnexin is used as a loading control. 20µg of total protein were loaded in each lane.
h) Comparative filter trap of poly-GA and poly-PR aggregates with GFP antibody reveals that loading 25-fold more total protein is necessary to detect any poly-PR from affected PR-Nes brains.

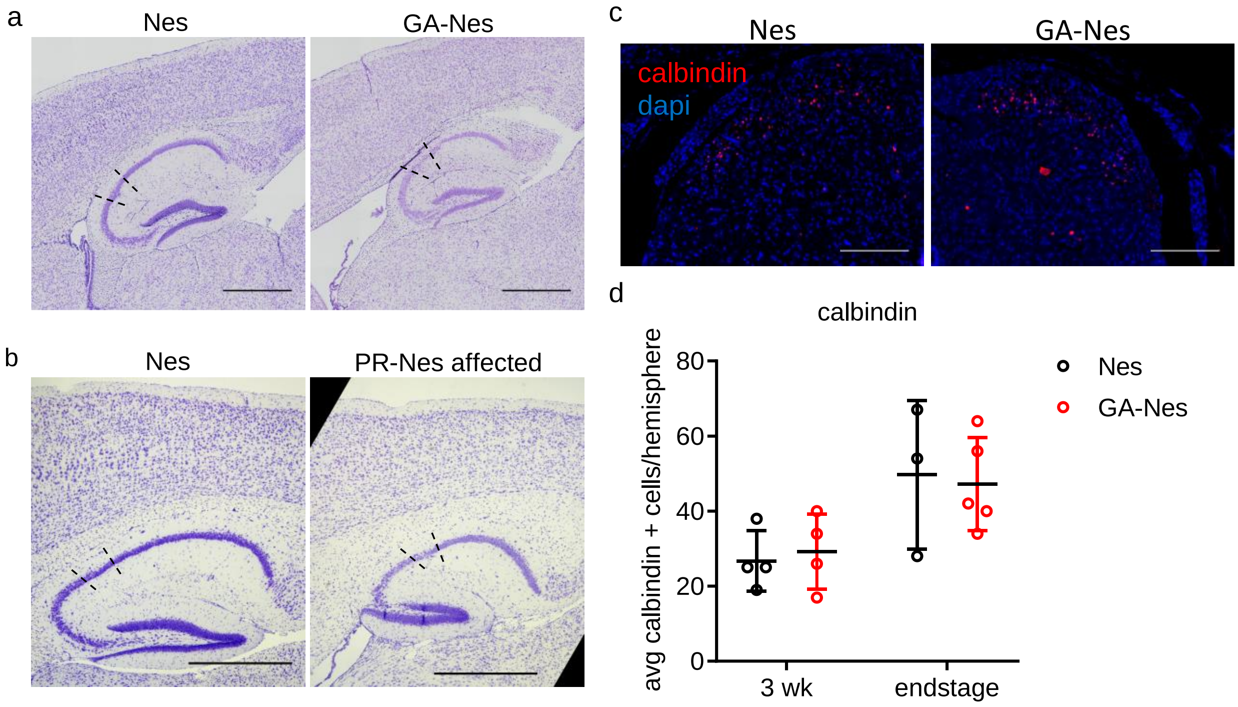

Fig. S2. Neuron loss in GA-Nes mice is restricted to specific neuronal populations

a) Representative Nissl stain of end stage GA-Nes mice and Nes control shows thinning of the CA fields but not the cortex (quantified in Fig. 2e). CA2 outlined by dashed lines. Scale bar = 500µm.

b) Representative Nissl stain of end stage affected PR-Nes mice shows no obvious thinning of neuronal layers in the hippocampus or cortex compared to Nes control (quantified in Fig. 2e). CA2 outlined by dashed lines. Scale bar = 500µm.

c) Representative images of calbindin positive interneurons (red) and DAPI (blue) in the dorsal hemisphere of the spinal cord of end stage GA-Nes and control.

d) No significant difference is detected in the average number of calbindin interneurons/hemisphere in the dorsal horn sampled every 1 mm through the spinal cord between Nes and GA-Nes mice at 3 weeks or end stage [Two-way ANOVA: interaction (age x genotype) $F(1,12)= 0.151$, ns $p=0.70$]. 3 week n=4/group, end stage Nes n=3, GA-Nes n=5.

a

Analysis	GA (n=3)	GA-Nes (n=4)	adj. p value
WBC (10^3/uL)	5.43±1.21	4.86±0.74	0.990
Lymphocytes (%)	68.60±4.07	72.30±1.94	0.953
Neutrophils (%)	7.83±2.25	6.05±3.65	0.990
Monocytes (%)	21.73±3.76	18.9±3.01	0.990
Eosinophiles (%)	1.83±0.38	2.7±1.30	0.990
Basophiles (%)	0.00±0.00	0.05±0.10	0.990
Red blood cells (10^6/uL)	7.75±0.49	7.73±0.61	0.990
Platelets (10^3/uL)	650.00±233.77	861.00±222.60	0.986
Hemoglobin (g/dL)	12.40±0.61	12.78±0.99	0.990
Hematocrit (%)	39.83±1.36	39.20±3.28	0.990
MCV (fL)	51.47±2.12	50.75±0.80	0.990
MCH (pg)	16.03±0.87	16.53±0.43	0.990
MCHC (g/dL)	31.13±0.65	32.60±0.64	0.281
RDW-CV (%)	18.07±2.84	17.73±3.74	0.990
mean platelet volume (fL)	6.50±0.69	6.0±0.14s	0.961
Platelet distribution width (fL)	6.00±1.11	5.15±0.17	0.953
Platelet large cell ratio (%)	4.73±3.75	1.72±0.66	0.953
Plateletcrit (%)	0.41±0.11	0.52±0.48	0.990

Fig. S3. Hematological analysis of GA-Nes mice does not indicate any systemic inflammation or other abnormalities that could explain muscle wasting

a) Analyzed hematological parameters are not significantly different between Nes and GA-Nes mice at 5 weeks of age when muscle wasting is already apparent. Adjusted p-values from multiple two-tailed t-tests were determined using the Holm-Sidak method.

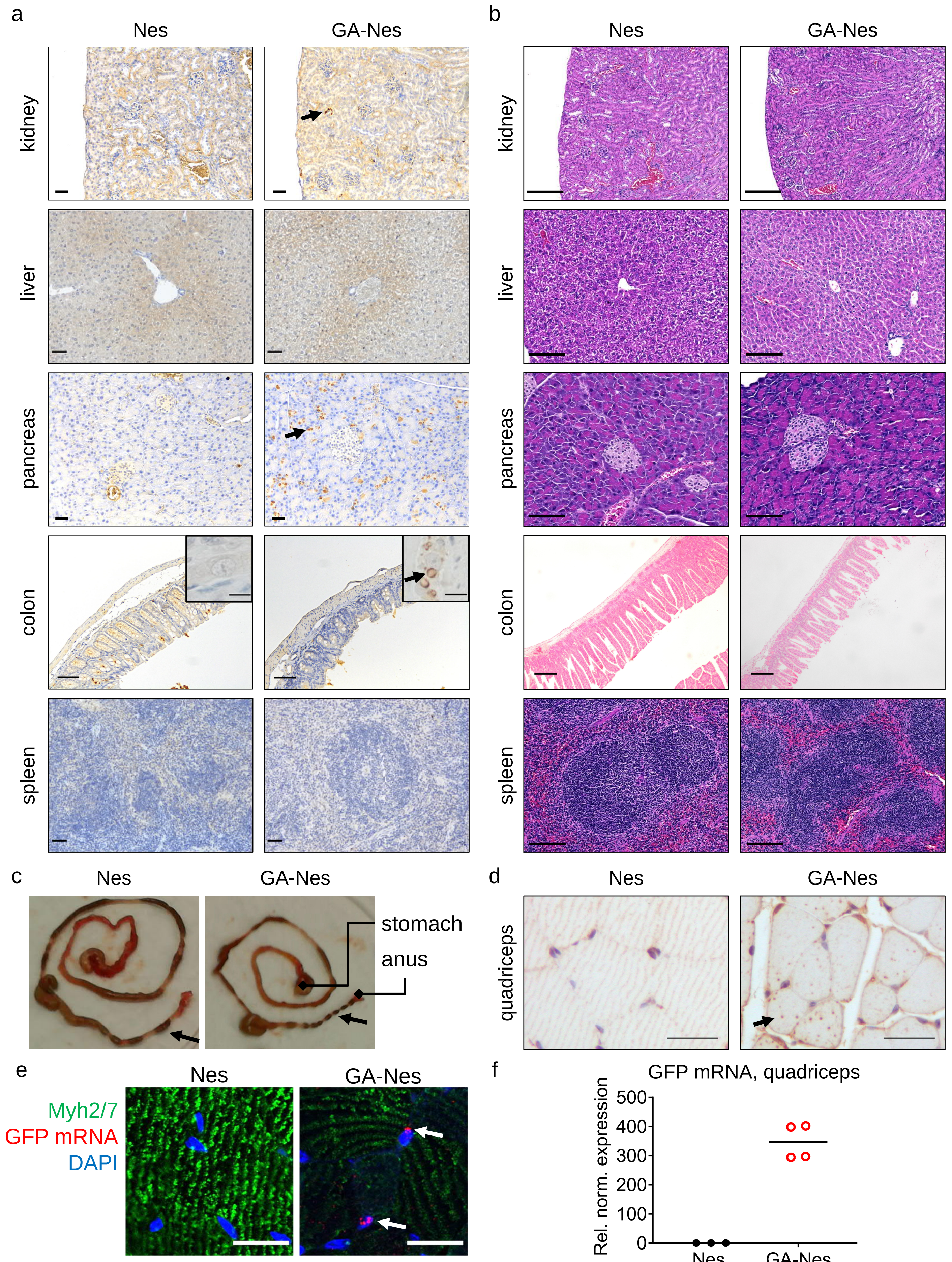

a
Nes
GA-Nes
kidney
liver
pancreas
colon
spleen
b
Nes
GA-Nes
kidney
liver
pancreas
colon
spleen
c
Nes
GA-Nes
stomach
anus
d
Nes
GA-Nes
quadriceps
e
Myh2/7
GFP mRNA
DAPI
Nes
GA-Nes
f
GFP mRNA, quadriceps
Rel. norm. expression
500
400
300
200
100
0
Nes
GA-Nes

Fig. S4

Fig. S4. Systematic analysis of peripheral organs in GA-Nes mice shows poly-GA expression in muscle, but no other peripheral mechanisms that could contribute to muscle wasting

a) Representative GFP-stained sections of the kidney, liver, pancreas, colon, spleen, and skeletal muscle show occasional poly-GA aggregates (arrows) in the kidney and pancreas, and more frequently in the neurons of the myenteric plexus in end stage GA-Nes mice, while other areas of the colon show non-specific staining also present in Nes control. Kidney, liver, pancreas, spleen scale bar =100µm, colon scale bar = 500µm, insets 10µm.

b) Representative H+E stained sections of the kidney, liver, pancreas, colon, spleen, and skeletal muscle show no signs of necrosis or gross pathological abnormality other than reduced size consistent with overall smaller GA-Nes mice compared to control littermates. Kidney, liver, pancreas, spleen scale bar=200µm, colon scale bar = 500µm.

c) Representative images of the intact digestive tract at euthanasia showed regularly spaced boli throughout the entire length and no impaction. No enlargement typical for peristalsis defects is seen.

d) Representative GFP-stained sections of the quadriceps show numerous aggregates in skeletal muscle of GA-Nes mice (arrow). Scale bar=50µm.

e) Representative images of FISH for GFP mRNA (red) shows the presence of poly-GA RNA at the nuclei of muscle cells in the quadriceps (Mh2/7). Scale bar=20µm.

f) qPCR analysis for GFP confirms transgene expression in quadriceps muscle of GA-Nes mice.

a

TDP-43 antibody (epitope)	Detected aggregates in GA-Nes mice	Source
1D3 (pS409/410)	No	Neumann *et al*. 2009
CAC-TIP-PTD-P02 (pS409/410)	No	Cosmo
CAC-TIP-PTD-M01 (p409/410)	No	Proteintech
3655 (pS409/410)	No	Chew *et al*. 2015
CAC-TIP-PTD-P05 (pS403/404)	No	Cosmo
(pT153/Y155)	No	Li *et al*. 2017
12892-1-AP (polyclonal C-terminal)	No	Proteintech
10782-2-AP (polyclonal N-terminal)	Yes	Proteintech
TAR5 6D6 (non-phospho 404-413)	Yes	Neumann *et al*. 2009
TAR5 2H4 (non-phospho 404-413)	Yes	Neumann *et al*. 2009

b

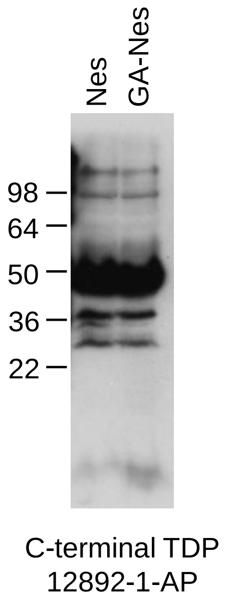

c

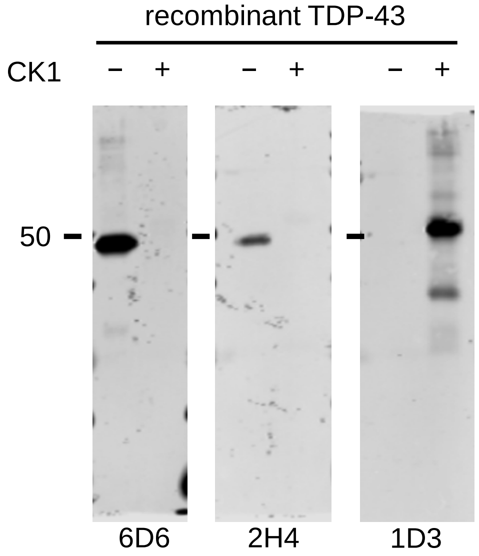

Fig. S5. Pathological TDP-43 phosphorylation or proteolytical processing is not detectable in GA-Nes mice

a) Table of TDP-43 antibodies tested including binding region and phosphorylation specificity and whether they detected TDP-43 nuclear aggregates in GA-Nes mice.

b) Immunoblot of C-terminal TDP-43 shows no additional cleavage products in homogenates from end stage GA-Nes cortex compared to Nes control.

c) Characterization of non-commercial antibodies from a) without previously published validation by immunoblot of recombinant TDP-43 with and without treatment with CK1 kinase (recTDP+CK1) to induce phosphorylation. The 6D6 and 2H4 antibodies recognize only unphosphorylated TDP-43, while the 1D3 antibody recognizes only phosphorylated TDP-43.

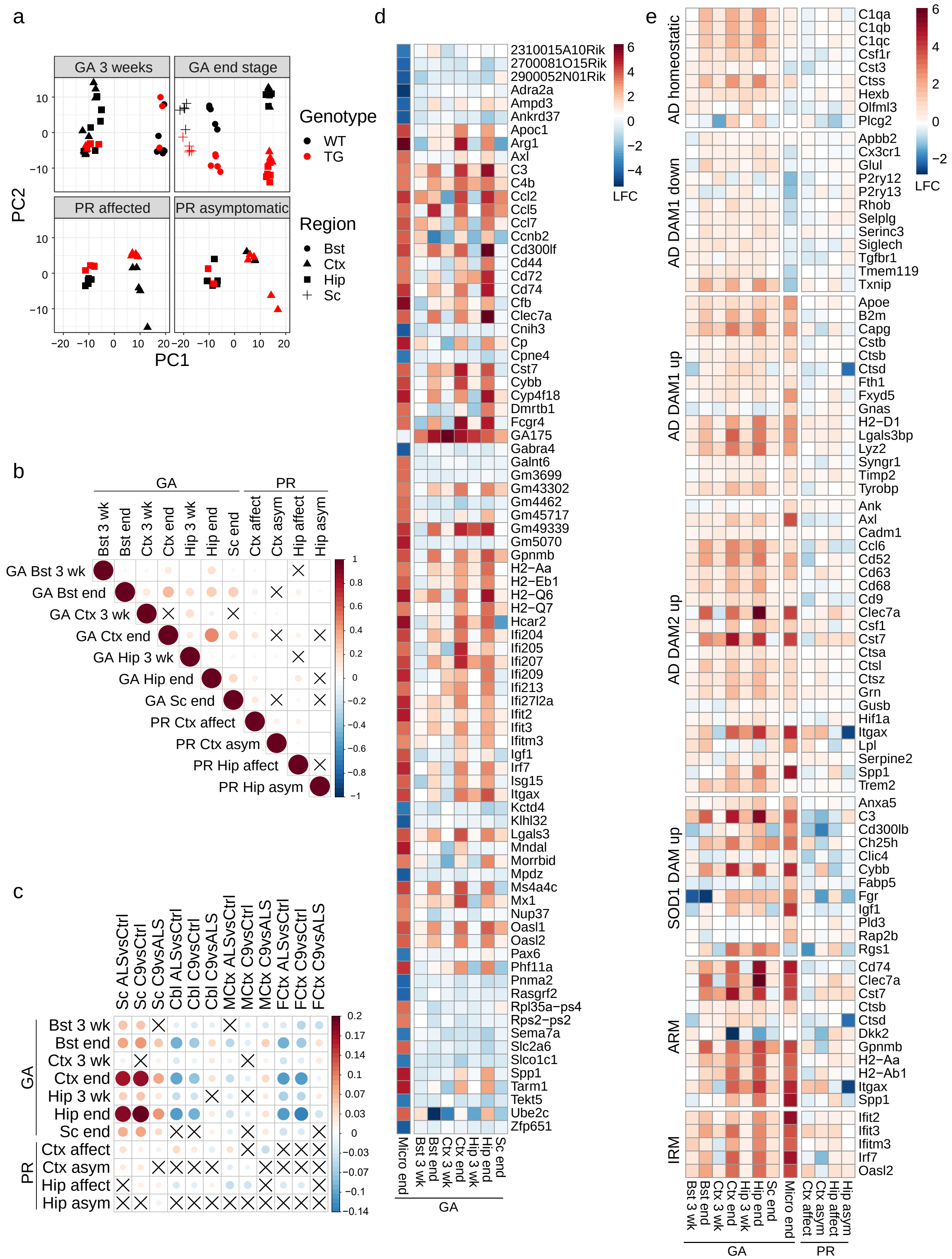

Fig. S6. Supporting RNAseq and protein data in GA-Nes and PR-Nes mice

a) Principle component analysis (PCA) of the RNAseq gene expression data of GA-Nes and control brainstem, cortex, hippocampus and spinal cord at 3 weeks and end stage shows dominant clustering by region at all ages (PC1) and clustering by genotype only at end stage (PC2). In contrast, PR-Nes genes cluster well by region and genotype in affected mice, but only by region in asymptomatic mice.
b) Correlation analysis of differential gene expression between different regions and time points of GA-Nes and PR-Nes mice. Color indicates Pearson's correlation coefficient. Crossed out comparisons are not significantly correlated.
c) Correlation analysis of differential gene expression between GA-Nes/PR-Nes CNS regions and human ALS patients (spinal cord, cerebellum, motor cortex, and frontal cortex) show strongest correlation between end stage GA-Nes mice and spinal cord from ALS patients. PR-Nes regions do not correlate strongly with any human ALS region. Color indicates Pearson's correlation coefficient. Crossed out comparisons are not significantly correlated.
d) Relative gene expression ($\log_2$ fold change, LFC) between GA-Nes and control mice filtered by genes with at least 10-fold expression differences between the genotypes in microglia. Transgene expression (GA175) is also listed to show lack of expression in microglia.
e) Analysis of signature genes for homeostatic and disease associated microglia subpopulations identified in Aβ and SOD1 mouse models [1]. Activated response microglia (ARM) and interferon response microglia (IRM) subpopulations were identified in Aβ expressing mice [2]. Heatmap of $\log_2$ fold changes (LFC) show predominant activation of the IRM subpopulation.

Supplemental Video 1 GA-Nes mouse (marked with red dot) shows abnormal gait and impaired motor function at 5 weeks compared to a control littermate (unmarked).
Supplemental Video 2 GA-Nes mouse at end stage shows severe muscle weakness and gait abnormalities. Video was taken just prior to euthanasia according to human endpoint criteria.
Supplemental Video 3 Ataxia and frequent loss of balance in an end stage affected PR-Nes mouse without any hindlimb clasping or apparent muscle weakness. Video was taken just prior to euthanasia according to human endpoint criteria.
Supplemental Video 4 Normal motor function in an asymptomatic PR-Nes mouse at 68 weeks.

Supplemental Table S1 Differential expression analysis of GA-Nes and PR-Nes samples from isolated microglia, brainstem, neocortex, hippocampus and spinal cord at 3 weeks or at end stage using DESeq2 as described in the methods. In column D -1 and +1 indicate significant genes ($p_{adjusted}$ p<0.05) and the direction of change.
Supplemental Table S2 Clustering of all genes differentially expressed in at least one comparison of GA-Nes and control littermates ($p_{adjusted}$ p<0.05).
Supplemental Table S3 Full Gene Ontology enrichment analysis for differential expressed genes from GA-Nes and PR-Nes samples in brainstem, neocortex, hippocampus and spinal cord at 3 weeks or at end stage. Up- and down-regulated genes were analyzed separately. Selected data is shown in Fig. 5b.
Supplemental Table S4 Differential expression analysis of TargetALS samples in cerebellum, frontal cortex, motor cortex and spinal cord using DESeq2 as described in the methods. ALSvsCtrl represents comparison of *C9orf72* negative ALS cases with controls, C9vsCtrl represents comparison of *C9orf72* positive ALS cases with controls and C9vsALS represents direct comparison of C9orf72 positive and negative ALS cases. In columns D-E -1 and +1 indicate significant genes ($p_{adjusted}$<0.05) and the direction of change.
Supplemental Table S5 Full Gene Ontology enrichment analysis for differential expressed genes from TargetALS patients in spinal cord (SC) and cerebellum (CBL) and genes concordantly expressed in GA-Nes mice. For patients, we compared *C9orf72*-negative ALS with healthy controls (ALSvsCtrl), *C9orf72*-positive ALS with healthy controls (C9vsCtrl) and *C9orf72*-postive with *C9orf72*-negative ALS cases (C9vsALS). Selected data is shown in Fig. 6a.

Supplemental References

1 Keren-Shaul H, Spinrad A, Weiner A, Matcovitch-Natan O, Dvir-Szternfeld R, Ulland TK, David E, Baruch K, Lara-Astaiso D, Toth B et al (2017) A Unique Microglia Type Associated with Restricting Development of Alzheimer's Disease. Cell 169: 1276-1290 e1217 Doi 10.1016/j.cell.2017.05.018
2 Sala Frigerio C, Wolfs L, Fattorelli N, Thrupp N, Voytyuk I, Schmidt I, Mancuso R, Chen WT, Woodbury ME, Srivastava G et al (2019) The Major Risk Factors for Alzheimer's Disease: Age, Sex, and Genes Modulate the Microglia Response to Abeta Plaques. Cell Rep 27: 1293-1306 e1296 Doi 10.1016/j.celrep.2019.03.099
